# Supplementary material for: GenNet framework: interpretable deep learning for predicting phenotypes from genetic data
Source: Commun Biol. 2021 Sep 17;4:1094. doi: 10.1038/s42003-021-02622-z (PMC8448759; doi:10.1038/s42003-021-02622-z)
Supplement: Supplementary file 2 — Supplementary Information [file 42003_2021_2622_MOESM2_ESM.pdf]

GenNet framework: interpretable deep learning for predicting phenotypes from genetic data

Supplementary Information

Table of contents

Supplementary Method 1. Simulations 3
1.1 Proof of concept 3
1.2 Synthetic data 4
1.3 Genotype data, simulated phenotype 4
Supplementary Note 2. Overview of the experiments 6
2.1 Gene networks 6
2.2 Pathway networks 7
2.2 GTEx expression networks 10
2.2 ImmGen expression networks 11
Supplementary Note 3. Sweden-Schizophrenia population-based case-control study 12
3.1 Gene network 13
3.2 Gene network with an L1 constraint on the weights 14
3.3 Pathway network 15
3.4 Gene-cell network & gene-GTEx networks 16
Supplementary Note 4. UK Biobank 19
4.1 Bipolar disorder 19
4.2 Skin color 20
4.3 Hair color: one versus all 21
4.4 Hair color: one vs one 24
4.5 Male balding pattern 25
4.6 Breast cancer 26
4.7 Asthma 27
4.8 Dementia 27
4.9 Coronary artery disease 28
4.10 Atrial fibrillation 28
4.11 Diabetes 29
Supplementary Note 5. Rotterdam Study 30
5.1 Eye color – blue vs rest 30
Supplementary Method 6. Estimating upper bound classification accuracy 31
6.1 Overview of the upper bound classification accuracies in this study 32
Supplementary Discussion 7. Deletion of predictive connections 33

Supplementary Discussion 8. Does prior knowledge improve performance? 34
Supplementary Note 9. GenNet architectures 36
9.1 All architectures: summary table 36
9.2 Prior knowledge 37
9.3 URLS 37
Supplementary Method 10. Regression 39
Supplementary References 40

Supplementary Method 1. Simulations

1.1 Proof of concept

This experiment is easily reproduced by running: https://tinyurl.com/y8hh8rul online[1].
The first experiment is a simple experiment designed to show the working principle and how to interpret the results. 200 SNPs are simulated by drawing from a binomial distribution. These SNPs are assigned to 10 genes. The first 5 are set to be causal for our phenotype while the last 5 function as controls. The sizes of the genes are varied to observe the effects of different sizes on the importance, for every causal gene there is a control gene with the same size. (Gene sizes = [50, 30, 10, 5, 5, 50, 30, 10, 5, 5]. The training, validation and test set consist of 10000, 2000 and 2000 subjects respectively (50 % cases vs 50% controls). To ensure equal importance of the genes (even with different gene size) only 1 SNP per gene is set to be causal. Therefore, there are 5 different subtypes of the disease each subtype prevalent in 1/5th of the simulated population.

This is a relatively simple problem and the neural network performs as expected with a near perfect score (AUC of 0.99) in the test set. It can be seen in figure 1 that the network identifies the first five genes to be causal and the last five control genes to be of insignificant importance. Inspection of the weights between SNPs and genes identifies the causal SNPs without any unambiguity for all causal genes. The direction of effect is easily derived by the sign of the weights. Subtypes of the simulated phenotype can be distinguished by activation patterns.

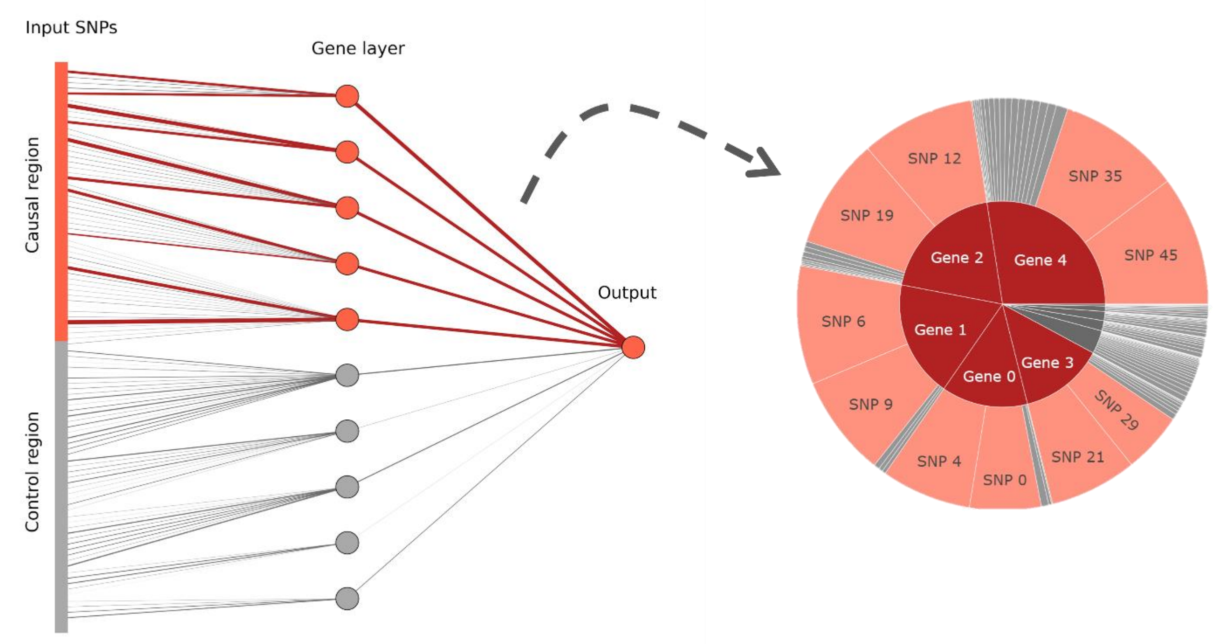

Supplementary Figure 1: A simple, non-linear proof of concept. In this simulation, each gene in the causal region has two causal SNPs that cause the simulated disease. The magnitude of the learned weight is represented by the line thickness (contributing causal connections in red, non-contributing control connections in grey). The right side shows how this converts to a sunburst plot.

1.2 Synthetic data

In this simulation the performance of the network is evaluated when heritability, polygenicity and training set sample size are varied. 100,000 SNPs are simulated for N subjects by drawing from a binomial distribution (n=2, p=0.3). Causal SNPs are selected randomly and assigned equal effect size. The number of causal SNPs is controlled by the polygenicity variable. Liability is calculated according to: $Liability = \sqrt[2]{h^2}g + q\sqrt[2]{1-h^2}$ with h^2 as heritability, g as genomic risk (effect size multiplied by genotype matrix) and with q as random noise.
Phenotype is based on a liability-threshold model with the threshold half a standard deviation above the mean liability.

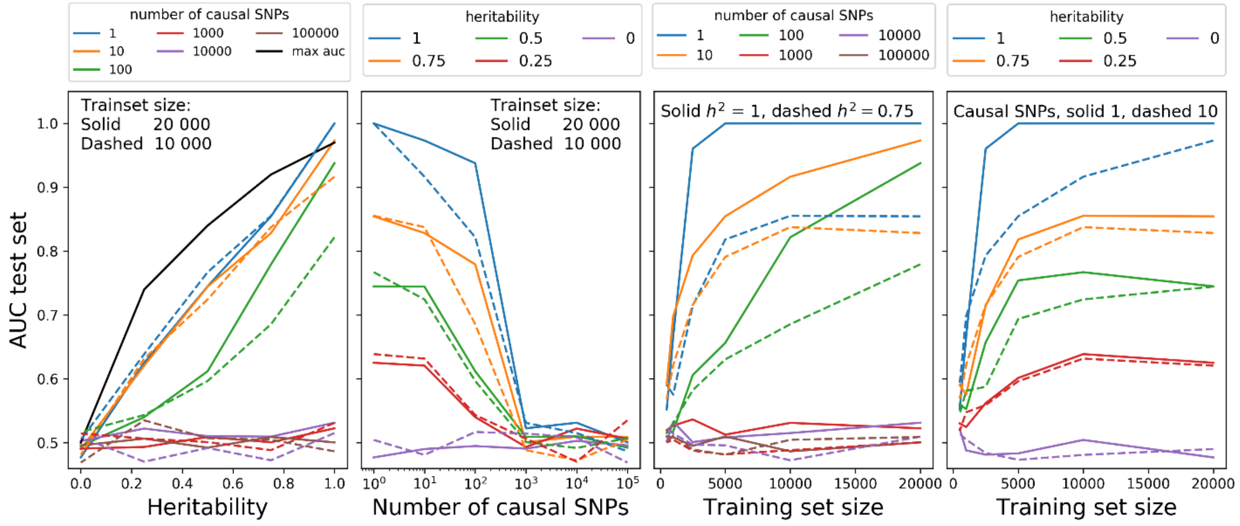

Supplementary Figure 2: Simulations with synthetic data showing the performance of GenNet expressed in the area under the curve for increasing levels of heritability, training set size and the number of causal SNPs (polygenicity). In total 540 simulations have been run with each 100,000 input variants In black the theoretical maximum of the AUC versus heritability.[2]

Supplementary Figure 2 gives an indication of the expected performance of a 3-layer network for different heritability, polygenicity and sample sizes. The indications are conservative, the network performed better in human genotype than in the simulated data. In genotype data the network obtained better performance for smaller sample sizes and for phenotypes with more causal SNPs than suggested by these simulations. This could be due to the absence of linkage disequilibrium in the simulated data or it could be an artifact of how the phenotype is constructed in the simulations.

1.3 Genotype data, simulated phenotype

The simulations are constructed from real data, the genotype matrix from the schizophrenia Sweden study served as a basis for this simulation. New labels were generated for each patient based on the following pseudo code:

Randomly select a set of **P** genes.
Randomly sample a number of SNPs in each gene (**N**).
Effect of causal SNPs: $\mathbf{E} = \frac{2}{\mathbf{NP}}$
Effect of non-causal SNPs: $\mathbf{E} = 0$

Phenotype risk = **E M** (Genotype matrix Sweden **M**)
Threshold for cases is the median phenotype risk, in order to obtain an equal case control ratio.

This experiment was designed to show the effect of LD blocks on the behavior of the neural network. Similar as seen in genome wide association studies, the found genes are not guaranteed to be causal.

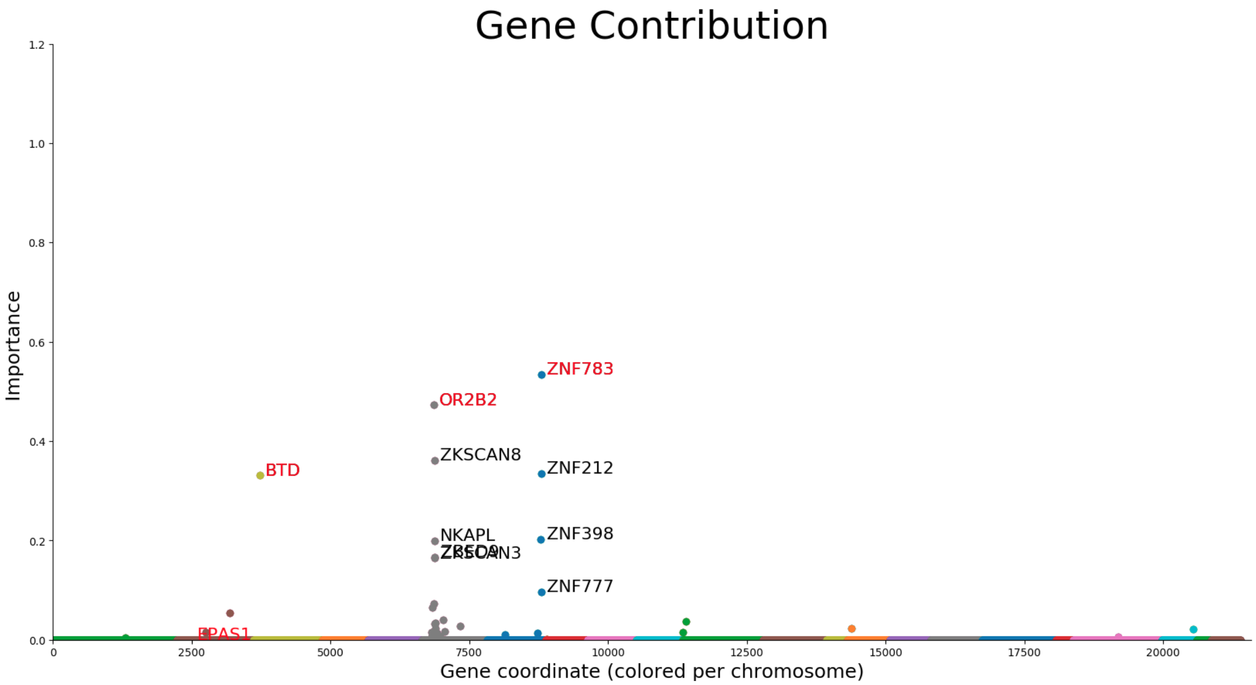

Supplementary Figure 3. Behavior of the Network for LD. Causal genes are in red, non-causal genes are in black. We can observe that the neural network tags SNPs similar to GWAS. The LD structure adds predictive value to non-causal genes due to the linkage disequilibrium.

Supplementary Note 2. Overview of the experiments

2.1 Gene networks

Dataset (type)	Number of input variants	Trait	Subjects & phenotype Class I	Class II	AUC LASSO (val)	AUC LASSO (test)	AUC GenNet (val)	AUC GenNet (test)	GenNet: top three most important genes
Rotterdam (genotype array)	113,241 (exonic) inputs of 16,628 genes	Eye color	4041 Blue	2250 Other	0.68	0.69	0.74	0.75	*HERC2*, *OCA2*, *LAMC1*
UK Biobank (exome)	6,986,636 input variants of 15,827 genes	Hair color	4501 Blond	4518 Other	0.62	0.61	0.65	0.66	*OCA2*, *TC2N*, *SLC45A2*
			15684 Dark brown	15918 Other	0.57	0.59	0.69	0.70	*OCA2*, *TC2N*, *SPIRE2*
			1734 Red	1727 Other	0.67	0.70	0.94	0.93	*MC1R*, SHOC2*, *DCTN3*
			16208 Light brown	16029 Other	0.61	0.62	0.62	0.62	*OCA2*, *TC2N*, *NOX4*
			3762 Black	3753 Other	0.85	0.82	0.83	0.81	*OCA2*, *RPL23AP87*, *SPIRE2*
		Skin color	1883 Fair	1894 Dark	0.97	0.98	0.98	0.98	*RPL23AP87*, *SMARCAD1*, *GLP1R*
		Male balding pattern	3454 Balding pattern 1	3454 Balding pattern 4	0.57	0.57	0.56	0.57	*NGEF*, *NKRD18B*, *SYNJ2*
		Atrial fibrillation	192 Cases	194 Controls	0.64	0.43	0.59	0.56	*** BRINP1*, *SORBS3*, *ELM0D3*
		Coronary Artery Disease	1563 Cases	1600 Controls	0.57	0.55	0.58	0.57	*** STARD7-AS1*, *VWC2L*, *NSD2*
		Bipolar disorder	343 Cases	347 Controls	0.56	0.59	0.56	0.60	*LINC00266-1*, *CSMD1*, *TCERG1L*
		Diabetes	2557 Cases	2555 Controls	0.55	0.54	0.57	0.54	***DNAH10*, *SNAR-I*, *PSMD13*
		Dementia	139 Cases	142 Controls	0.55	0.58	0.65	0.61	*RPL23AP87*, *CTNNA3*, *LINC01003*

		Allergies	10242 Cases	10187 Controls	0.51	0.51	0.53	0.52	***AC025039.1, AC004052.1, VPS45*
		Breast cancer	1070 Cases	1082 Controls	0.53	0.52	0.56	0.52	*RPL23AP87, LINC00266-1, HPSE2*
		Asthma	4229 Cases	4214 Controls	0.53	0.51	0.57	0.55	*HLA-DQB1, HCG9, LINC00266-1*
Sweden (exome)	1,288,701 input variants of 21,390 genes	Schizophrenia	4969 Cases	6245 Controls	0.65	0.65	0.73	0.74	*ZNF773, PCNT, DYSF*

*Supplementary Table 1. Summary of the experiments and results in this study for the simplest network in our framework that contains the input SNPs, the gene layer and the output layer. Genes were annotated using ANNOVAR.[3] Manhattan plots for gene importance can be found in Supplementary Materials 3,4 & 5. *MC1R was not present in gene annotations but was identified by linkage disequilibrium. **Many genes contributed to the prediction without clear separation between genes, see Supplementary Materials 4.*

2.2 Pathway networks

**Dataset (type)**	**Trait**	**Subjects & phenotype**		**AUC GenNet pathway (val)**	**AUC GenNet pathway (test)**	**GenNet: top three most important pathways**		
		**Class I**	**Class II**			**Global**	**Mid**	**Local**
Rotterdam (genotype array)	Eye color	4041 Blue	2250 Other	0.52	0.50	Organismal Systems (78.4%), Cellular Processes (17.9%), Human Diseases (3.0%) Systems (11.7%)	Digestive system (72.6%), Transport and catabolism (17.9%), Circulatory system (5.7%)	Pancreatic secretion (59.1%), Vitamin digestion and absorption (13.3%), Autophagy - animal (9.5%)
UK Biobank (exome)	Hair color	4501 Blond	4518 Other	0.57	0.58	Organismal Systems (70.2%), Environmental Information Processing (21.4%), Cellular Processes (3.5%)	Endocrine system (30.1%), Signal transduction (19.6%), Immune system (14.8%)	Adrenergic signaling in cardiomyocytes (4.1%), Olfactory transduction (3.9%), Insulin signaling pathway (3.6%)
		15684 Dark brown	15918 Other	0.55	0.56	Human Diseases (37.2%), Metabolism (27.2%), Cellular Processes (26.1%)	Metabolism of cofactors and vitamins (26.7%), Substance dependence (17.9%), Transport and catabolism (17.2%)	Thiamine metabolism (26.2%), Endocytosis (17.0%), Parkinson disease (9.6%)
		1734 Red	1727 Other	0.77	0.77	Genetic Information Processing (87.4%), Human	Replication and repair (83.4%), Translation (2.5%), Infectious diseases:	Fanconi anemia pathway (79.7%), Homologous recombination

					Diseases (9.6%), Metabolism (2.8%)	Bacterial (2.3%)	(2.2%), Legionellosis (2.0%)
	16208 Light brown	16029 Other	0.57	0.57	Environmental Information Processing (55.1%), Human Diseases (28.3%), Cellular Processes (8.6%)	Signal transduction (54.3%), Infectious diseases: Bacterial (7.8%), Cancers: Overview (5.9%)	MAPK signaling pathway (8.1%), Rap1 signaling pathway (7.2%), Calcium signaling pathway (6.1%)
	3762 Black	3753 Other	0.78	0.76	Organismal Systems (46.9%), Human Diseases (29.7%), Cellular Processes (16.9%)	Endocrine system (18.6%), Cellular community - eukaryotes (12.7%), Nervous system (10.9%)	Axon guidance (5.0%), Focal adhesion (3.7%), Tight junction (3.1%)
Breast cancer	1070 Cases	20545 Controls	0.56	0.51	Human Diseases (57.1%), Organismal Systems (27.1%), Metabolism (8.5%)	Infectious diseases: Viral (16.6%), Cancers: Overview (16.4%), Cancers: Specific types (14.1%)	Pathways in cancer (6.5%), Metabolic pathways (4.2%), Gastric cancer (3.4%)
Diabetes	2557 Cases	2555 Controls	0.54	0.54	Environmental Information Processing (43.5%), Organismal Systems (26.1%), Human Diseases (18.3%)	Signal transduction (40.5%), Nervous system (8.4%), Cancers: Specific types (6.9%)	Ras signaling pathway (7.9%), cAMP signaling pathway (7.2%), MAPK signaling pathway (5.9%)
Atrial fibrillation	192 Cases	194 Controls	0.63	0.57	Organismal Systems (39.6%), Environmental Information Processing (21.0%), Human Diseases (18.6%)	Signal transduction (11.2%), Transport and catabolism (10.7%), Immune system (9.7%)	Cytokine-cytokine receptor interaction (4.4%), Endocytosis (3.8%), Axon guidance (2.9%)
Coronary Artery Disease	1563 Cases	1600 Controls	0.56	0.54	Environmental Information Processing (29.5%), Organismal Systems (23.7%), Cellular Processes (15.8%)	Signal transduction (27.7%), Cellular community - eukaryotes (8.8%), Immune system (5.5%)	PI3K-Akt signaling pathway (4.6%), Focal adhesion (3.7%), Tight junction (2.5%)
Bipolar disorder	343 Cases	347 Controls	0.55	0.47	Organismal Systems (76.9%), Cellular Processes (16.4%), Metabolism (5.7%)	Endocrine system (46.5%), Transport and catabolism (16.4%), Immune system (11.8%)	Melanogenesis (32.9%), Thyroid hormone signaling pathway (12.2%), Phagosome (9.0%)
Dementia	139 Cases	142 Controls	0.58	0.55	Human Diseases (39.8%), Environmental Information Processing (26.5%), Organismal Systems (22.8%)	Signal transduction (22.2%), Cancers: Overview (13.3%), Endocrine system (7.3%)	Pathways in cancer (5.6%), PI3K-Akt signaling pathway (4.1%), Proteoglycans in cancer (2.8%)
Male balding pattern	3454 Balding pattern 1	3454 Balding pattern 4	0.55	0.54	Organismal Systems (34.6%), Human Diseases (22.7%), Metabolism (20.8%)	Nervous system (9.7%), Global and overview maps (8.9%), Transport and catabolism (8.3%)	Metabolic pathways (8.7%), Endocytosis (4.3%), Axon guidance (4.1%)
Asthma	4229 Cases	4214 Controls	0.54	0.51	Genetic Information Processing (52.3%),	Folding, sorting and degradation (41.5%), Cardiovascular	Ubiquitin mediated proteolysis (22.0%), Protein processing in endoplasmic

						Metabolism (23.6%), Human Diseases (23.2%)	diseases (12.6%), Amino acid metabolism (12.2%)	reticulum (11.8%), RNA transport (10.8%)
Sweden (exome)	Schizophrenia	4969 Cases	6245 Controls	0.67	0.68	Human Diseases (30.8%), Organismal Systems (26.7%), Genetic Information Processing (26.5%)	Infectious diseases: Viral (27.3%), Endocrine system (16.6%), Folding, sorting and degradation (13.7%)	Human papillomavirus infection (11.7%), Ubiquitin mediated proteolysis (10.0%), Ribosome biogenesis in eukaryotes (4.4%)

Supplementary Table 2: Neural networks build with gene and KEGG pathway[4] information. The pathway information is hierarchical with 3 levels: global, mid and local. In this table the top three pathways for each level are displayed in terms of relative contribution. Interactive plots for all phenotypes can be found at: https://github.com/ArnovanHilten/GenNet_paper_plots

2.2 GTEx expression networks

Dataset (type)	Number of input variants	Trait	Subjects & phenotype		AUC GenNet GTEx (val)	AUC GenNet GTEx (test)	GenNet: top three most important cell types
			Class I	Class II			
Rotterdam (genotype array)	113,241 (exonic) inputs of 16,628 genes	Eye color	4041 Blue	2250 Other	0.76	0.76	Esophagus Gastroesophageal Junction (10.1%), Colon Sigmoid (9.5%), Esophagus Muscularis (7.5%)
UK Biobank (exome)	6,986,636 input variants of 15,827 genes	Hair color	4501 Blond	4518 Other	0.67	0.64	Brain Nucleus accumbens (basal ganglia) (4.1%), Brain Putamen (basal ganglia) (3.6%), Artery Tibial (3.5%)
			15684 Dark brown	15918 Other	0.60	0.62	Brain Frontal Cortex (BA9) (4.3%), Brain Hippocampus (3.6%), Brain Caudate (basal ganglia) (3.5%)
			1734 Red	1727 Other	0.76	0.76	Brain Caudate (basal ganglia) (4.1%), Esophagus Muscularis (4.1%), Brain Amygdala (3.9%)
			16208 Light brown	16029 Other	0.61	0.61	Brain Putamen (basal ganglia) (3.9%), Brain Hypothalamus (3.7%), Brain Cerebellar Hemisphere (3.6%)
			3762 Black	3753 Other	0.80	0.80	Artery tibial (4,0%), artery aorta (3.7%), esophagus gastroesophageal junction (3.7%),
		Diabetes	2557 Cases	2555 Controls	0.55	0.56	Brain Cortex (4.4%), Esophagus Gastroesophageal Junction (4.4%), Artery Tibial (4.2%)
		Atrial fibrillation	192 Cases	194 Controls	0.65	0.49	Esophagus Muscularis (4.2%), Brain Cerebellum (4.1%), Artery Aorta (3.6%)
		Coronary Artery Disease	1563 Cases	1600 Controls	0.55	0.54	Brain Caudate (basal ganglia) (4.2%), Brain Putamen (basal ganglia) (4.1%), Brain Amygdala (4.0%)
		Bipolar disorder	343 Cases	347 Controls	0.56	0.58	Brain Caudate (basal ganglia) (4.6%), Brain Hippocampus (4.6%), Brain Cerebellar Hemisphere (4.3%)
		Dementia	139 Cases	142 Controls	0.52	0.41	Brain Putamen (basal ganglia) (4.3%), Brain Caudate (basal ganglia) (4.2%), Brain Amygdala (4.1%)
Sweden (exome)	1,288,701 input variants of 21,390 genes	Schizophrenia	4969 Cases	6245 Controls	0.66	0.66	Uterus (4.2%), Colon Sigmoid (3.8%), Breast Mammary Tissue (3.7%)

Supplementary Table 3: Neural networks build with genes and tissue expression (GTEx) only the significantly different expressed genes are connected to their tissues Finucane et al. (2018)[5] *to*

ensure an interpretable network. Interactive plots for all phenotypes can be found at:

https://github.com/ArnovanHilten/GenNet_paper_plots

2.2 ImmGen expression networks

Dataset (type)	Trait	Subjects & phenotype		AUC GenNet GTEx (val)	AUC GenNet GTEx (test)	Most important immunological cell types
		**Class I**	**Class II**			
Rotterdam (genotype array)	Eye color	4041 Blue	2250 Other	0.50	0.50	Tgd.vg2+24ahi.Th (5.3%), T.DPsm.Th (5.0%), Ep.5wk.MEChi.Th (4.1%)
UK Biobank (exome)	Hair color	4501 Blond	4518 Other	0.50	0.48	proB.FrA.BM (5.5%), MF.Microglia.CNS (4.9%), SC.CDP.BM (4.7%)
		15684 Dark brown	15918 Other	0.50	0.50	T.8SP24int.Th (0.6%), MF.F480hi.ctrl.PC (0.6%), DN.SLN.CFA.d6.v2 (0.6%)
		1734 Red	1727 Other	0.49	0.47	DC.8+.MLN (2.1%), GN.BM (2.1%), DC.8-4-11b-.MLN (1.8%)
		16208 Light brown	16029 Other	0.48	0.50	FRC.SLN (0.6%), Tgd.vg5-.act.IEL (0.6%), MEChi.GFP+.Adult.KO (0.6%)
		3762 Black	3753 Other	0.79	0.78	T.8SP24int.Th (0.8%), T.4Mem44h62l.LN (0.8%), T.4Mem.LN (0.8%)
	Male balding pattern	3454 Balding pattern 1	3454 Balding pattern 4	0.50	0.51	GN.Bl.v2 (1.4%), DC.103+11b-.Lv (1.4%), DC.8-4-11b+.Sp (1.3%)
	Atrial fibrillation	192 Cases	194 Controls	0.53	0.41	Ep.5wk.MEClo.Th (0.8%), T.4.Pa.BDC (0.8%), Ep.MEChi.Th (0.8%)
	Coronary Artery Disease	1563 Cases	1600 Controls	0.57	0.53	Tgd.vg2+24ahi.Th (0.8%), DC.103-11b+F4/80lo.Kd (0.8%), Tgd.vg2+.Sp (0.8%)
	Bipolar disorder	343 Cases	347 Controls	0.54	0.57	FRC.Cad11.WT.v2 (0.8%), T.4Mem.Sp (0.8%), CD19Control (0.8%)
	Dementia	139 Cases	142 Controls	0.54	0.57	FRC.Cad11.WT.v2 (0.8%), DN.SLN.v2 (0.8%), preB.FrD.BM (0.8%)
Sweden (exome)	Schizophrenia	4969 Cases	6245 Controls	0.73	0.74	FRC.MLN (1.2%), DN.SLN.v2 (1.1%), SC.LT34F.BM (1.1%)

Supplementary Table 4: Neural networks build with genes and Immunological Genome Project data[6]. Only the significantly different expressed genes are connected to their tissues Finucane et al. (2018)[5] to ensure an interpretable network. Interactive plots for all phenotypes can be found at: https://github.com/ArnovanHilten/GenNet_paper_plots

Supplementary Note 3. Sweden-Schizophrenia population-based case-control study

All networks applied on the Sweden Schizophrenia Case Control study are trained with similar parameters, found by optimizing the performance on the validation set for the 'gene network'. The batch size was chosen to be 200, the optimizer Adam (learning rate of 0.0006), and with batch normalization after every tangent hyperbolicus activation. All networks are trained on a single GPU (Nvidia Geforce 1080 GTX) and converged within 3 hours.

**Network**	**AUC val. set**	**AUC test set**	**Accuracy test set**	**Source**
Gene Network	0.73	0.74	0.68	Annovar gene annotations
Gene Network L1	0.75	0.74	0.68	Annovar gene annotations
Gene -Pathway	0.67	0.68	0.61	Kegg pathway annotations
Gene-Tissue expr.	0.66	0.66	0.60	GTEx tissue expr.
Gene-Cell. expr.	0.74	0.75	0.68	FUMA

Supplementary Table 5. Overview of the performance for different networks. Area Under the Curve (AUC), Precision, Accuracy and F1 score for the different types of networks. The theoretical maximum accuracy is ~ 72%. The dataset was split 60/20/20 in training validation and with respect to the ratio of cases and controls. All results are reported on the test set with the optimum decision threshold determined by the ROC curve of the validation set.

3.1 Gene network

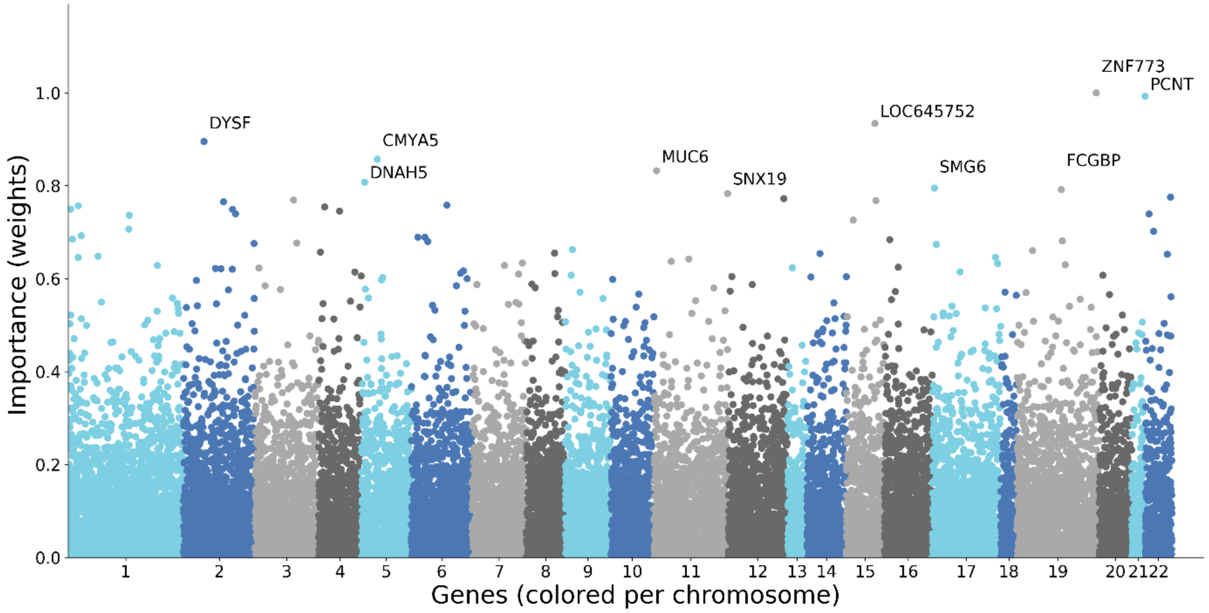

Supplementary Figure 4. Gene Importance for Schizophrenia predictions. Area under the curve for this model was 0.73 in the validation set and 0.74 in the test set. This was the best run of a series of experiments run with the same parameters. These models obtained a mean AUC of 0.70 ± 0.018 in the validation set and 0.72 ± 0.016 in the test set.

3.2 Gene network with an L1 constraint on the weights

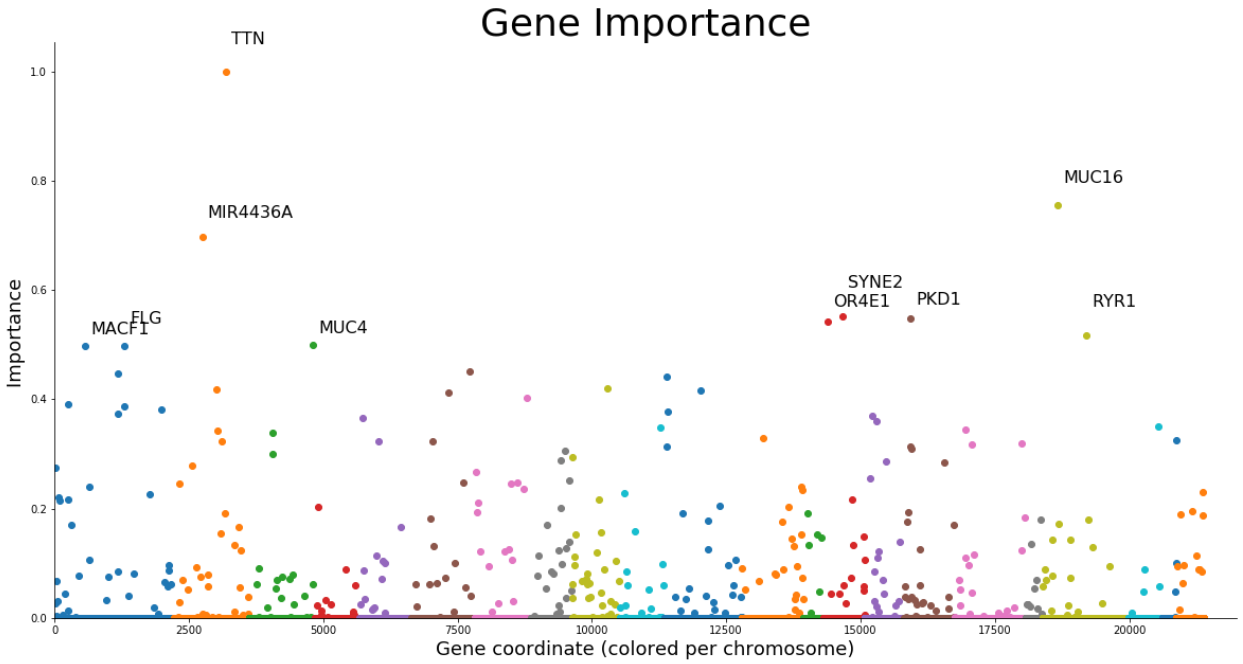

Supplementary Figure 5. Similar to lasso logistic regression, the network can be forced to focus on only the most important genes by adding L1 regularization, easing interpretation while maintaining performance (AUC of 0.74). The network is forced to focus only on the most important genes, leaving most genes with a near zero weight. As a consequence, larger genes are selected, such as *TTN* and *MUC16*. *MIR4436A* is therefore interesting since this is a micro-RNA of 63 base pairs.

3.3 Pathway network

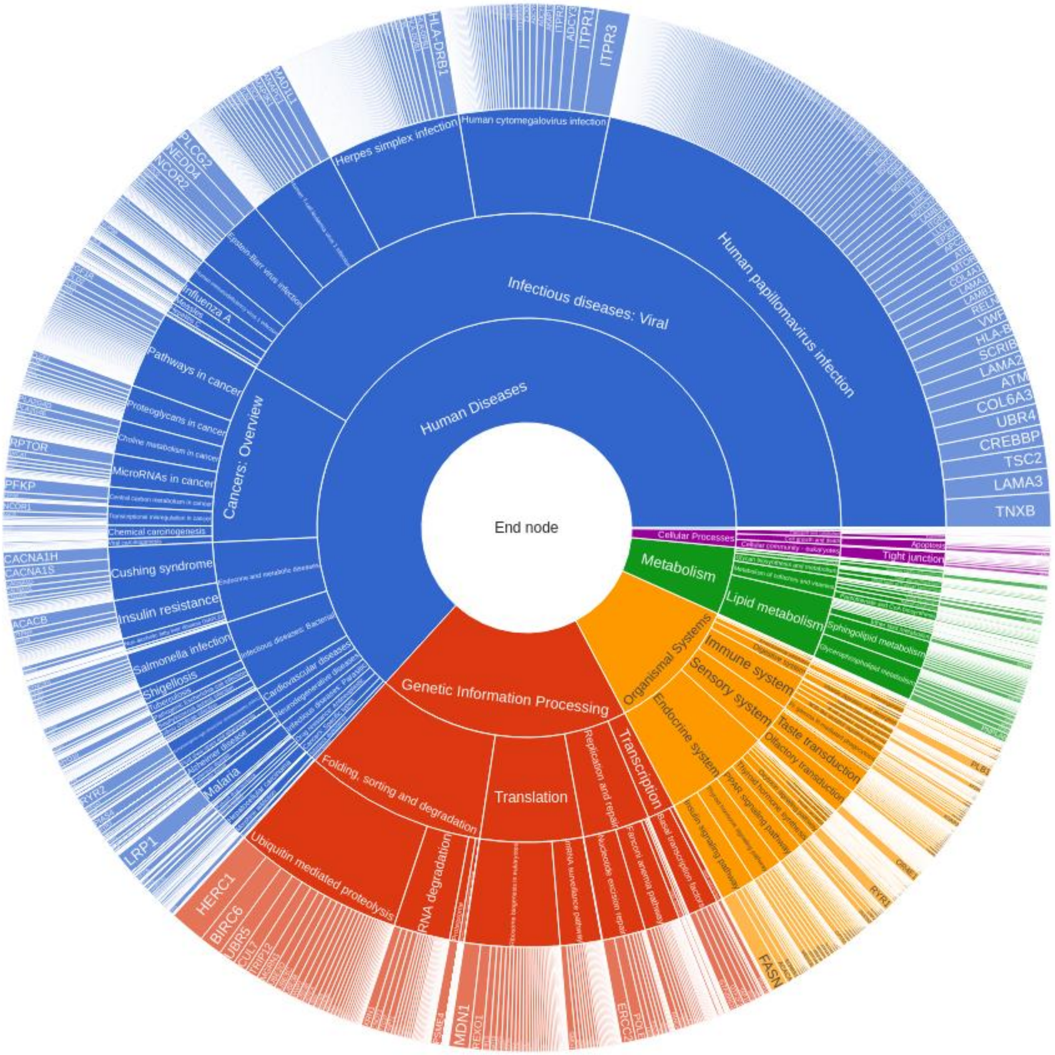

Supplementary Figure 6. The gene-pathway network achieved an AUC of 0.68 in the test set with only 7204 genes annotated by the KEGG database, leaving more than 14 000 genes unconnected. The performance of this network could thus be artificially inflated due to the same LD structure, leaking information of unconnected genes without a pathway. Pathway nodes are not as objectively and uniquely defined as gene nodes (see section 2.4). Nonetheless, pathway annotations could possibly regulate the network further to assign high weights to important pathways, genes and SNPs, guiding the network to select only disease relevant entities. This would ease interpretability, avoid tagging and will definitely increase performance. Moreover, pathway annotations allow us to create deeper networks, carrying more information about how;

from important SNPs to genes, genes to pathways and from pathways to the eventual trait everything relates to each other with respect to the trait.

3.4 Gene-cell network & gene-GTEx networks

There are multiple variations of these networks depending on the threshold used for defining connections (i.e., what is the level of expression required to start defining connections?).
An interesting point of research and a concern for the interpretability is the uniqueness of the connections required to be still interpretable. In the tissue and cell expression the connections are not as unique as in the gene layer, where overlap between inputs for neurons is rare. To avoid an arbitrary threshold the weights of the connections could be initialized with the level of expression. However, there is no guarantee that such a trained network would be interpretable.
To create more uniquely defined nodes, we chose to group and connect only the significantly associated genes per tissue or cell type. For this we used the approach and resources found in Finucane et al (2018)[5]. However, interpretability is still not guaranteed and further research is necessary to confirm interpretability.
Tutorials and examples for creating networks with cell and tissue expression are available on GitHub.

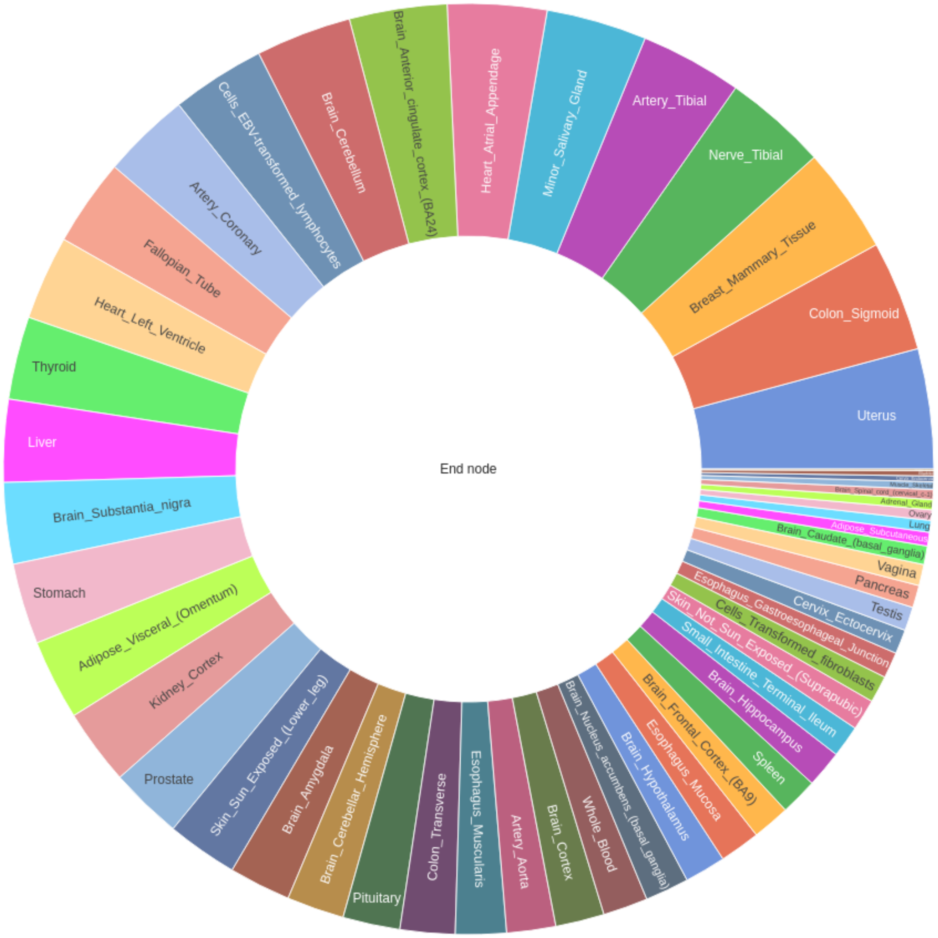

Supplementary Figure 7: Sunburst of the tissues and their relative importance using the GTEx tissue expression layer.

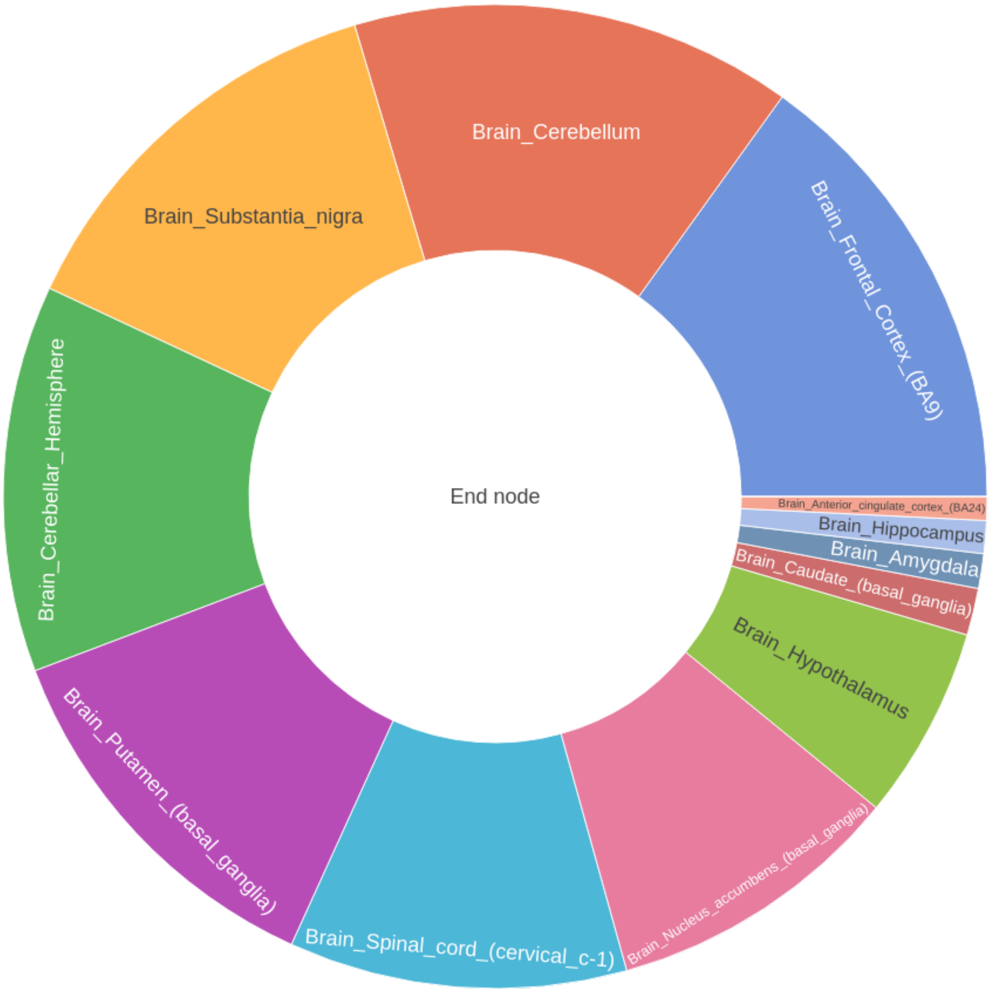

Supplementary Figure 8: Sunburst of the brain tissues and their relative importance using the GTEx brain expression layer.

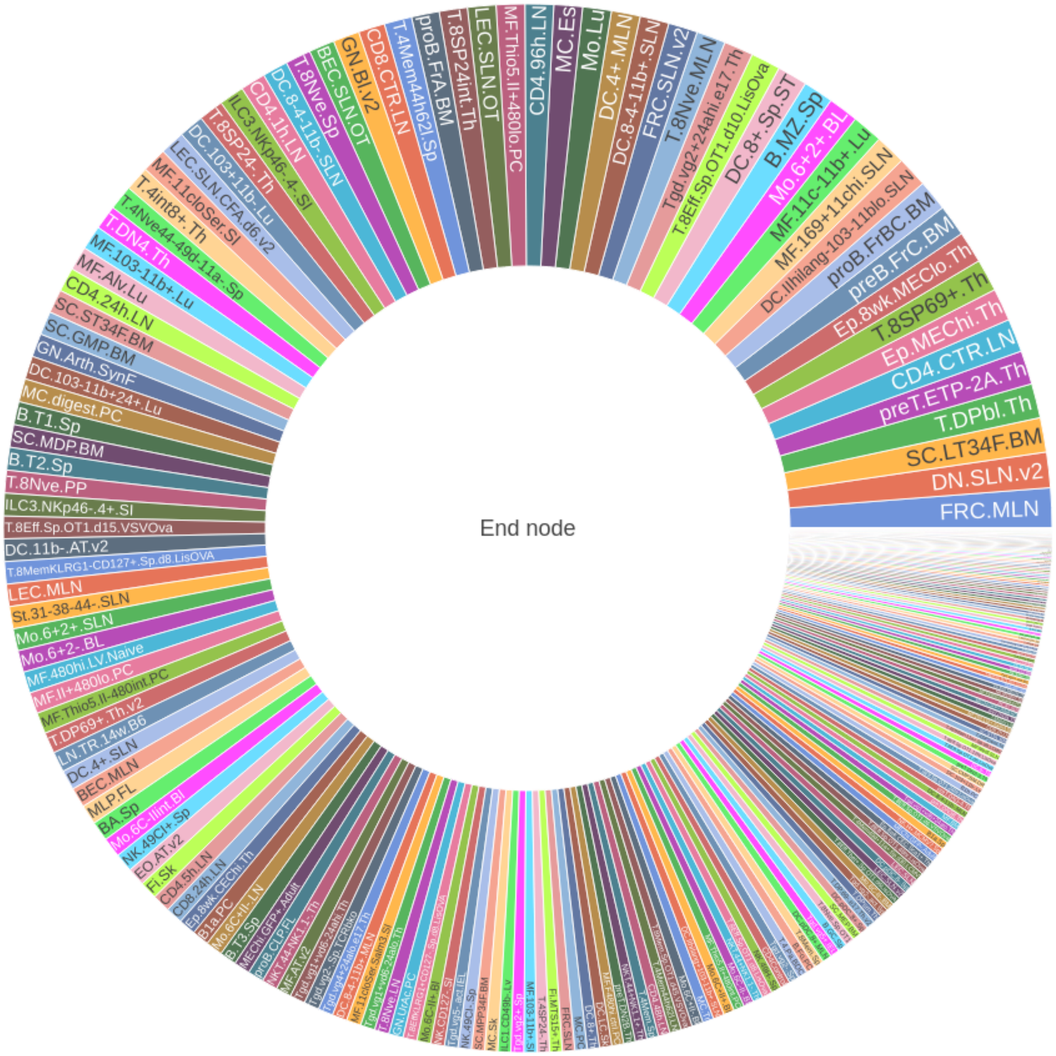

Supplementary Figure 9: Sunburst of the immune cell types and their relative importance using the ImmGen *layer. We used the preprocessed t-statistics made available from Finucane et al. (2018)* [5] *Raw data can be obtained from (*http://www.immgen.org/ *)*[6]

Supplementary Note 4. UK Biobank

4.1 Bipolar disorder

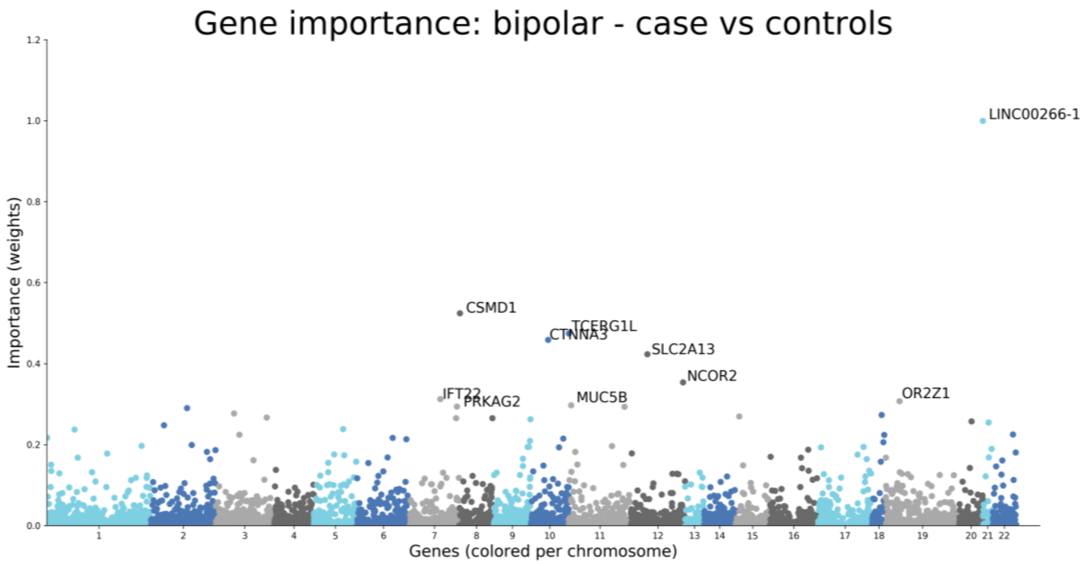

Supplementary Figure 10. Gene contribution for the prediction of bipolar disorder. *LINC00266-1* (Long Intergenic Non-Protein Coding RNA 266-1) on chromosome 22 is the greatest contributor.

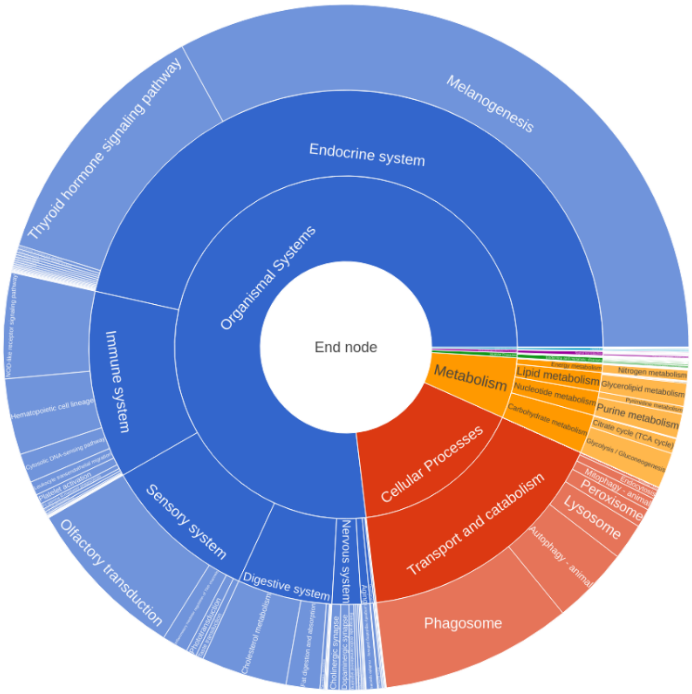

Supplementary Figure 11. Pathway contribution for the prediction of bipolar disorder.

4.2 Skin color

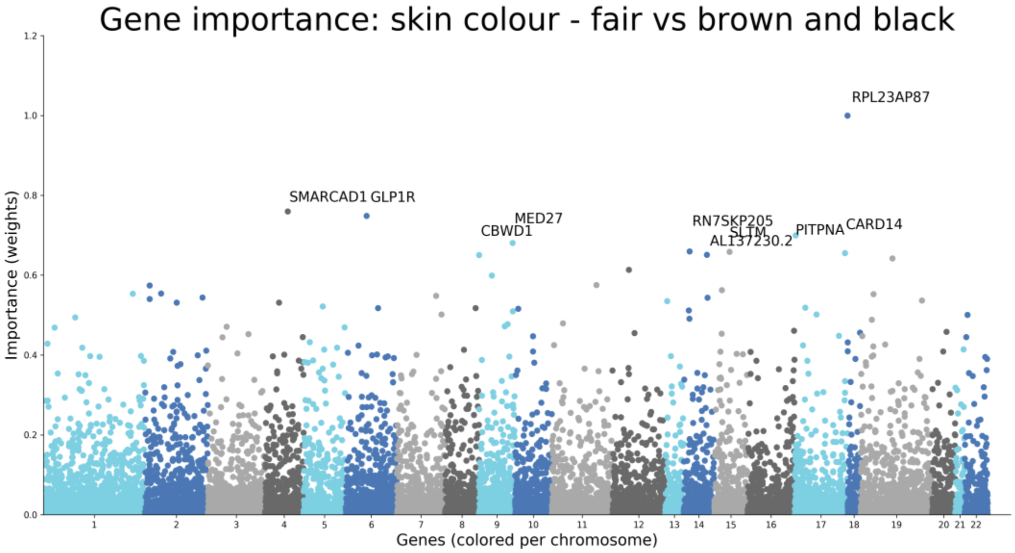

Supplementary Figure 12. The network to predict skin color obtains a near perfect score (AUC of 0.98 in the validation and test set) for distinguishing between a fair or a dark skin color Predicting skin color (fair vs brown and black) is close to predicting ethnicity. The predictions for skin color can serve as a good example why interpretability is a necessity for reliable predictions: interpretation of the network showed that the predictions are based on other factors such as ethnic background rather than genes related to pigmentation and skin color. Neural networks have better predictive capabilities than regular linear methods and can therefore identify and exploit biases in the dataset more easily. While using neural networks we should thus pay even more attention to confounders. *SMARCAD1* is associated with not having fingerprints.[7]

4.3 Hair color: one versus all

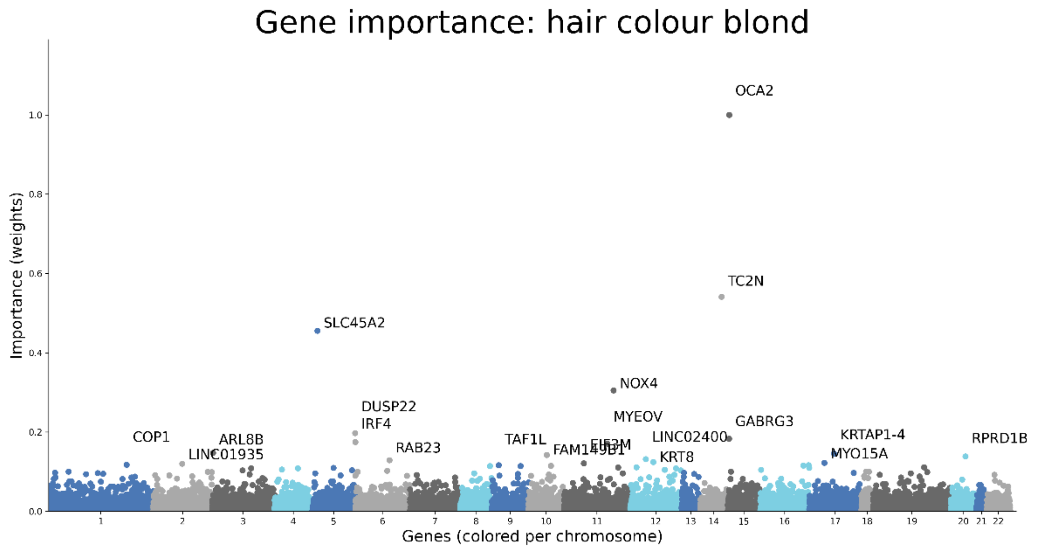

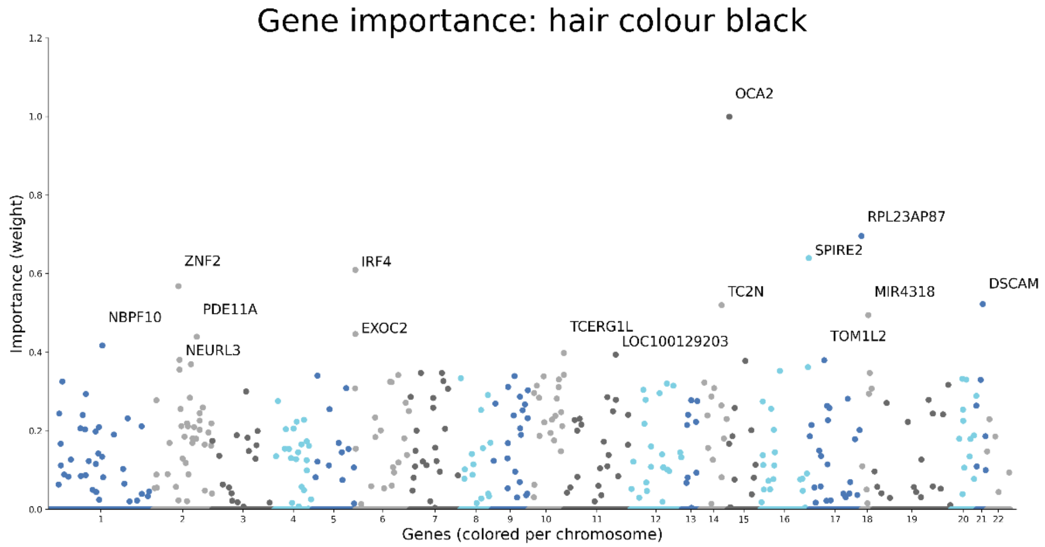

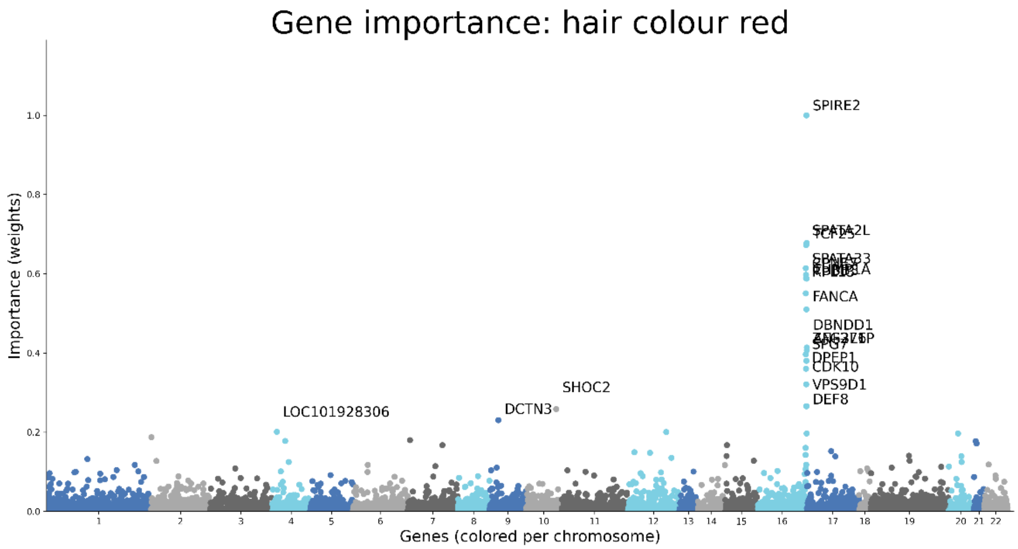
Gene importance: hair colour red
Importance (weights)
Genes (colored per chromosome)
SPIRE2
FANCA
DBNDD1
VPS9D1
DEF8
CDK10
SHOC2
DCTN3
LOC101928306

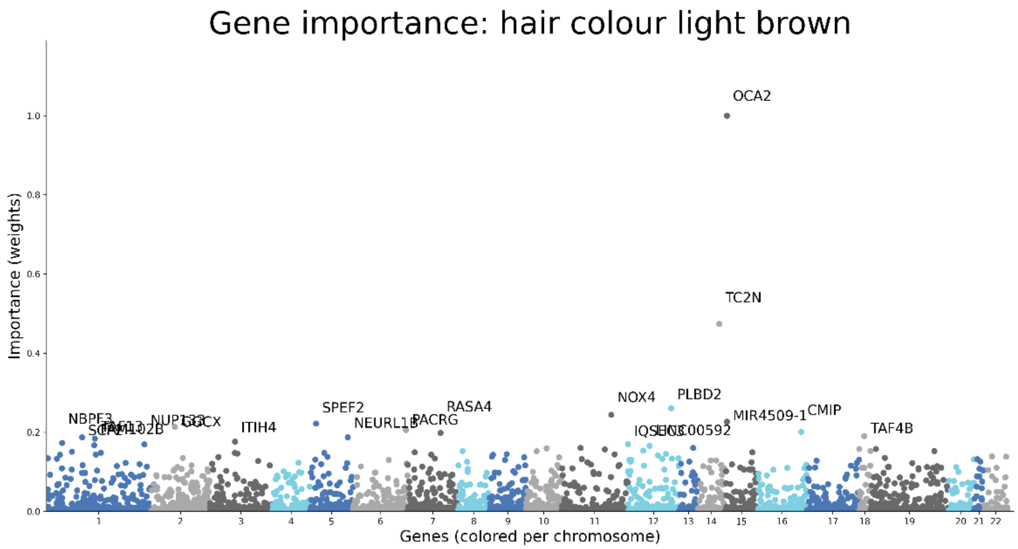
Gene importance: hair colour light brown
Importance (weights)
Genes (colored per chromosome)
OCA2
TC2N
NOX4
PLBD2
CMIP
MIR4509-1
TAF4B
SPEF2
RASA4
NEURL1B
PACRG
ITIH4
NBPF3

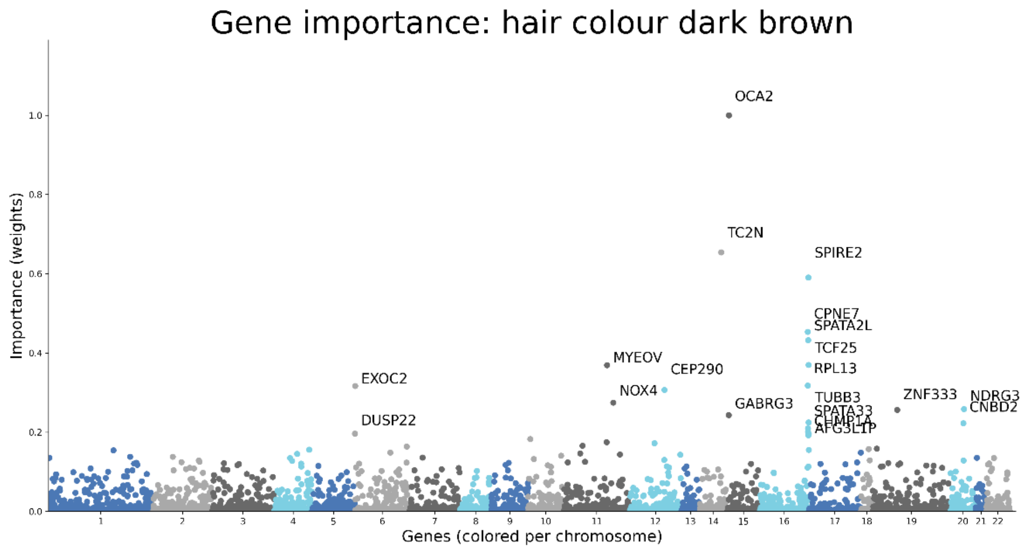

Supplementary Figure 13 to 17: The region on chromosome 16 contains the *MC1R* gene, a well-known gene that is associated with red hair color. Even though this gene was not present in the annotations, LD structure and the interpretability of the network allowed us to identify this gene. *OCA2* is a known gene, a melanin precursor, *EXOC2* has been identified before by Han et al. (2008).[8]

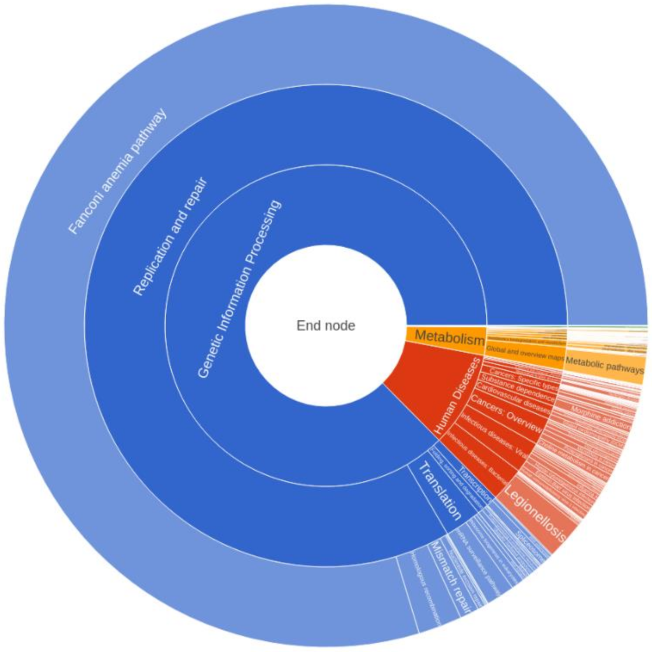

Supplementary Figure 18: Red hair color was dominated by the Fanconi anemia pathway.

4.4 Hair color: one vs one

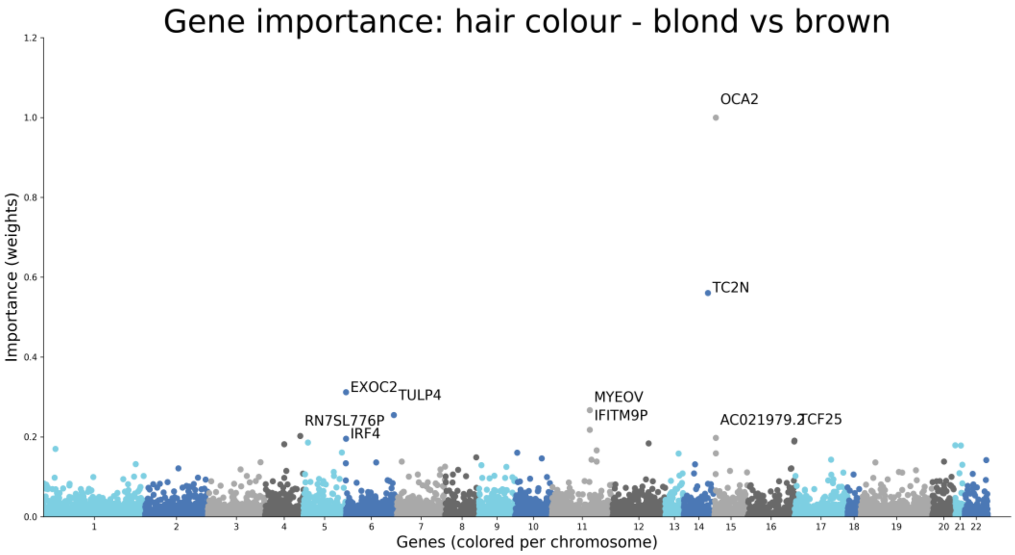

Supplementary Figure 19. *OCA2* is a known gene, a melanin precursor, *EXOC2* has been identified before by Han et al. (2008).[8]

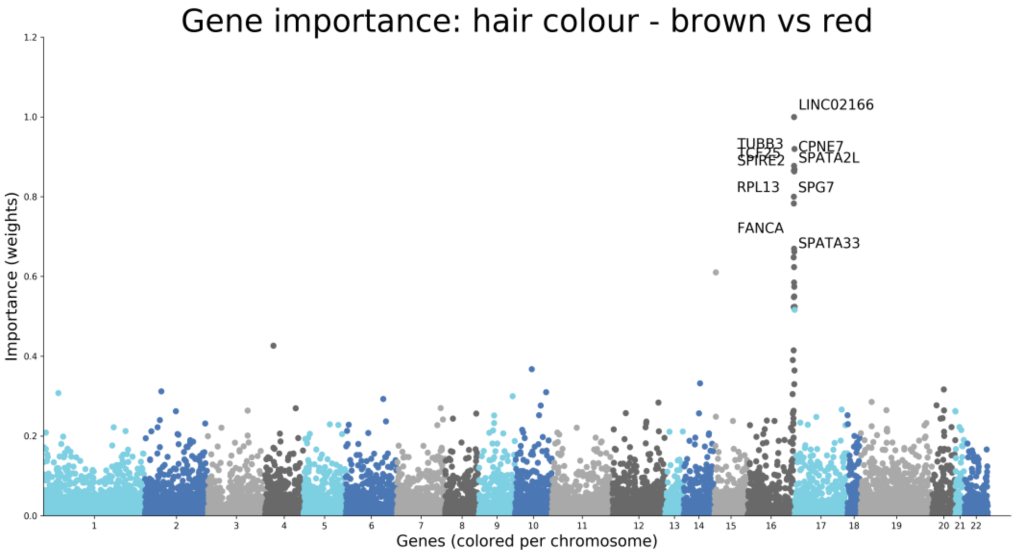

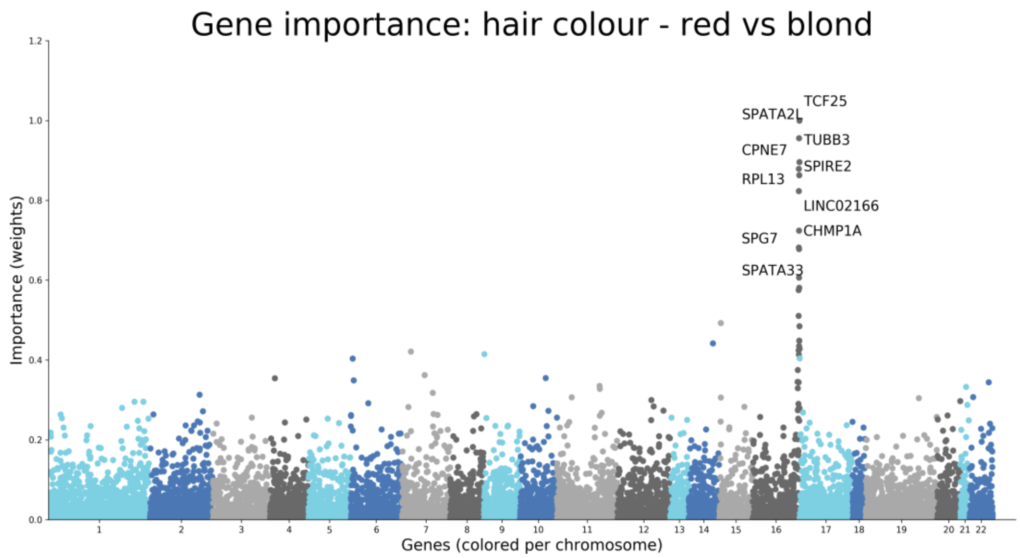

Supplementary Figure 20 & 21: Brown versus red and red versus blond hair. This region on chromosome 16 contains the *MC1R* gene, a well-known gene that is associated with red hair color. Even though this gene was not present in the annotations, LD structure and the interpretability of the network allowed us to identify this gene.

4.5 Male balding pattern

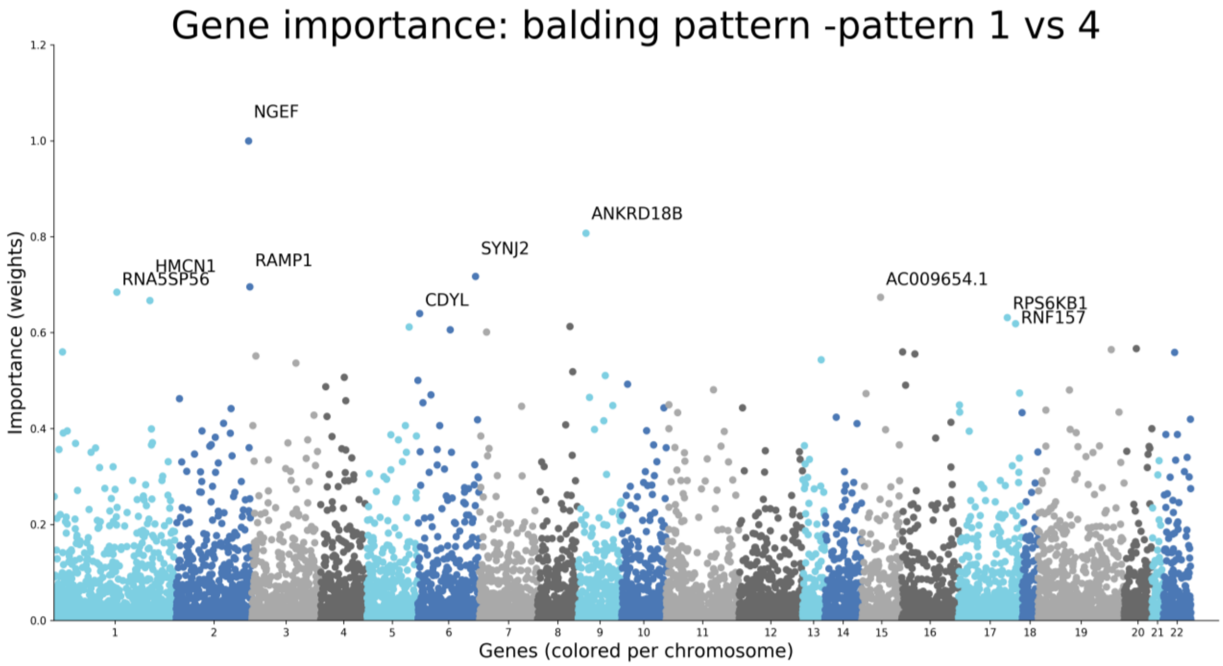

Supplementary Figure 22. Manhattan plot for genes important for the network for separating between balding pattern 1 and 4.

4.6 Breast cancer

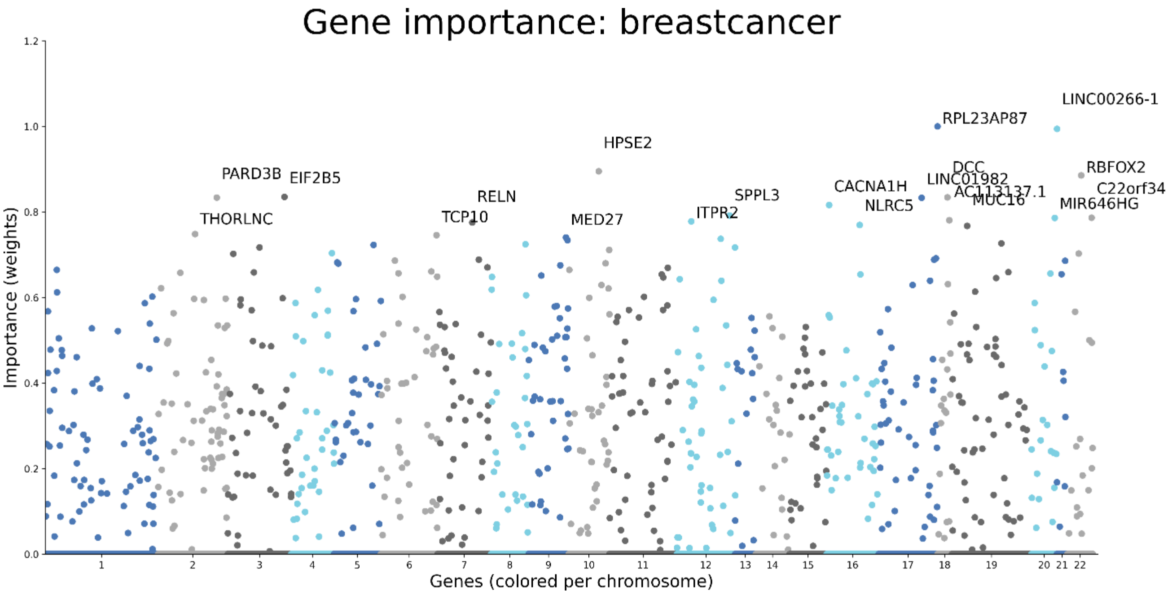

Supplementary Figure 23. Manhattan plot for genes important for the network for separating between cases and controls.

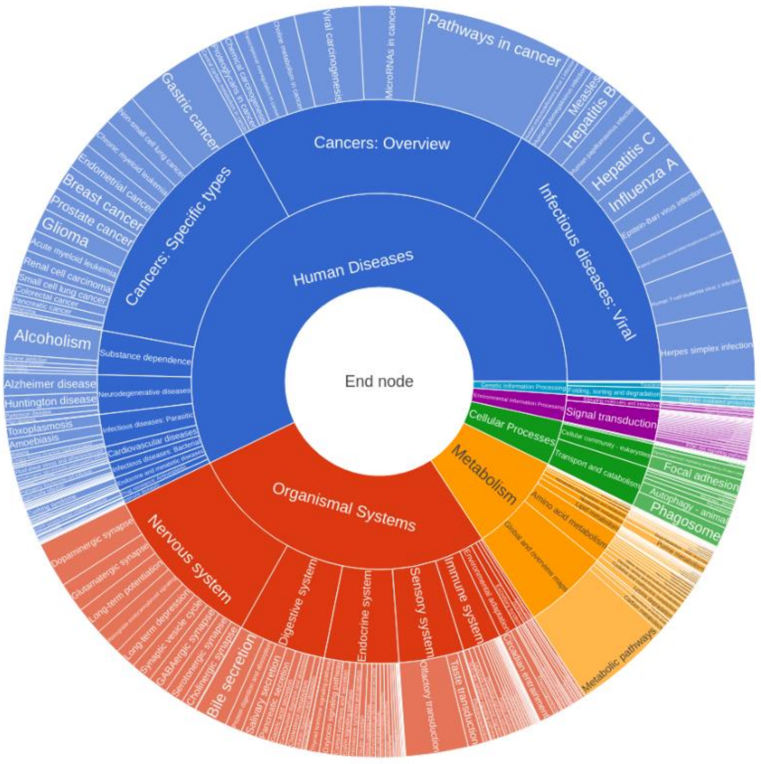

Supplementary Figure 24. Sunburst plot of pathways for cancers. Predictive performance for the pathway network was very low with 0.51 in the test set, 0.56 in the validation set.

4.7 Asthma

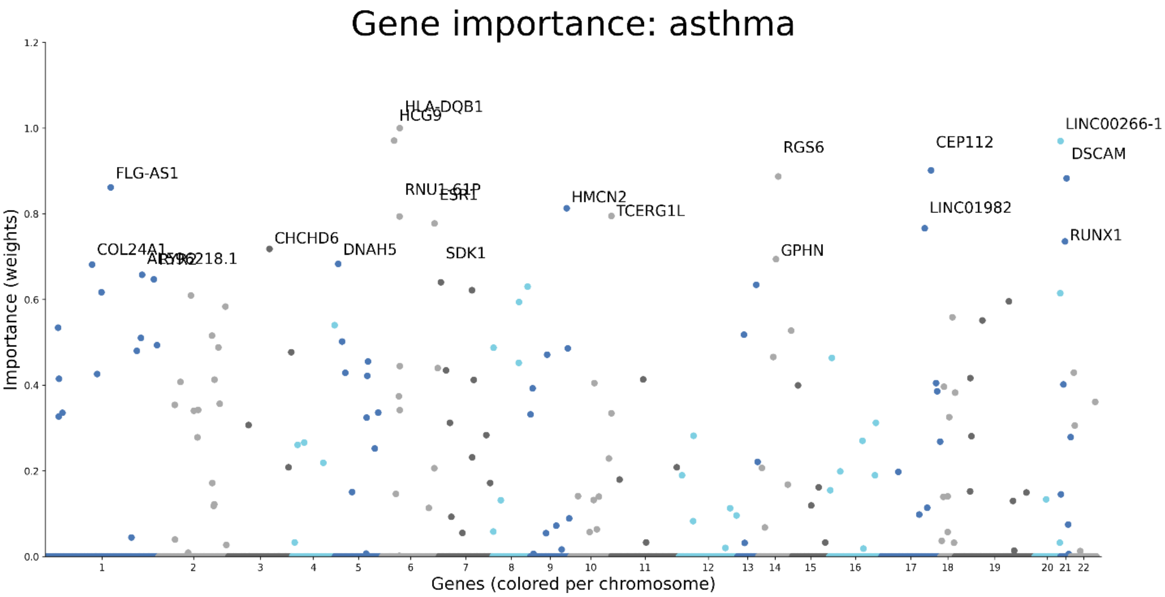

Supplementary Figure 25. Manhattan plot for genes important for the network for separating between cases and controls for asthma.

4.8 Dementia

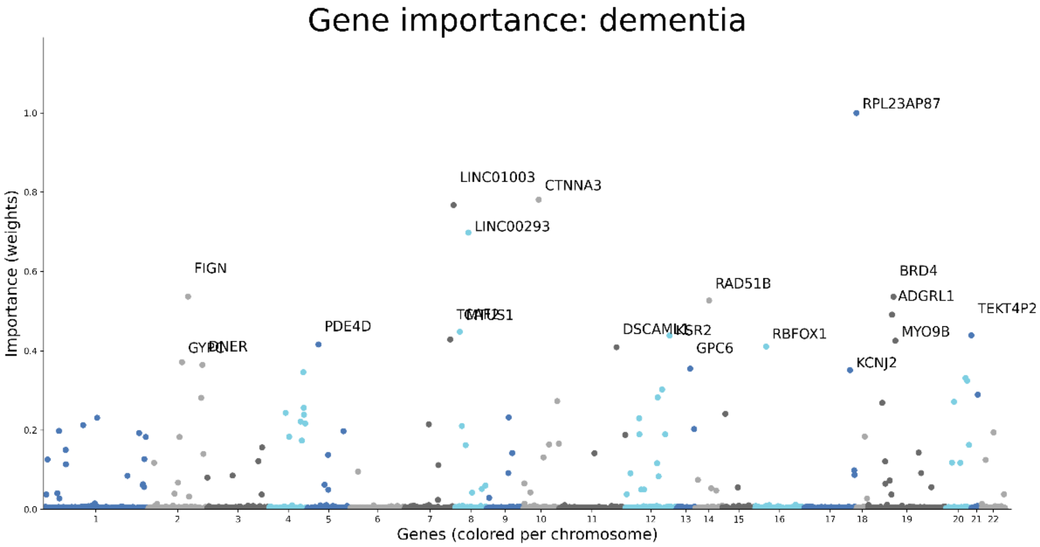

Supplementary Figure 26. Manhattan plot for genes important for the network for separating between cases and controls for dementia. APOE got assigned a (normalized) weight of 0.08

4.9 Coronary artery disease

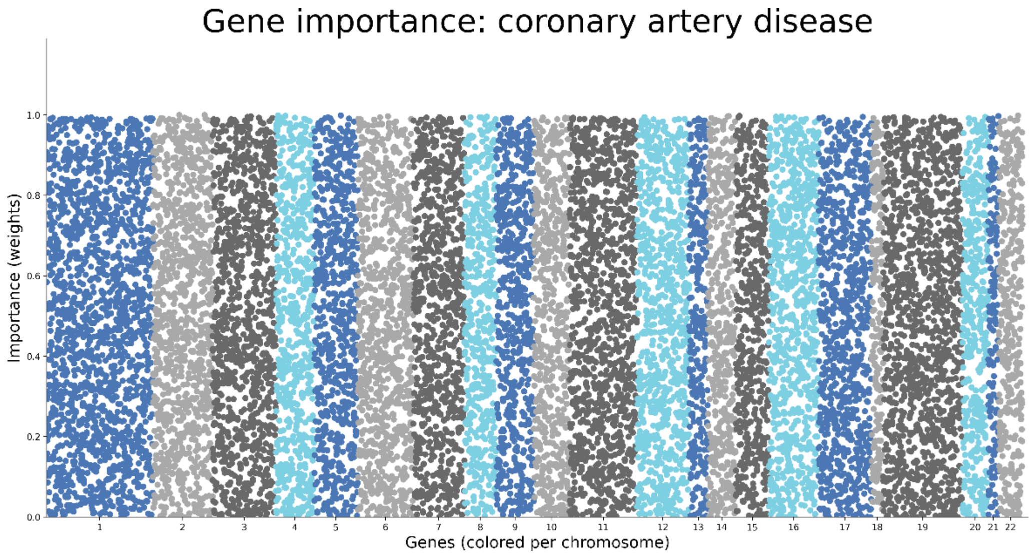

Supplementary Figure 27. Manhattan plot for genes important for the network for separating between cases and controls for coronary artery disease. The predictive performance was poor; 0.56 in the test set and 0.58 in the validation set and we suspect that we are underpowered. More data will most likely improve the predictive performance and therefore the interpretation.

4.10 Atrial fibrillation

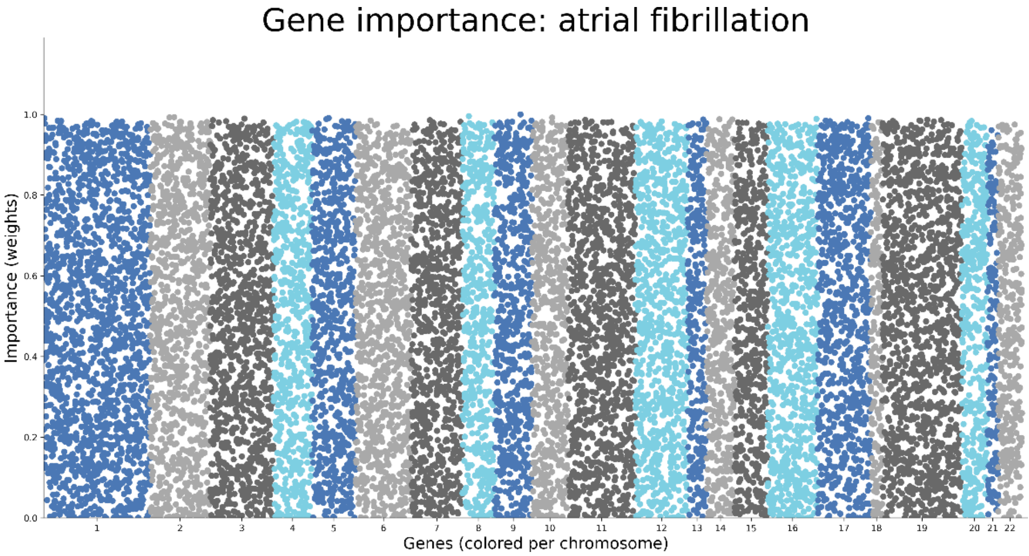

Supplementary Figure 28. Manhattan plot for genes important for the network for separating between cases and controls for atrial fibrillation. Similarly, to coronary artery disease we suspect we need a larger sample size.

4.11 Diabetes

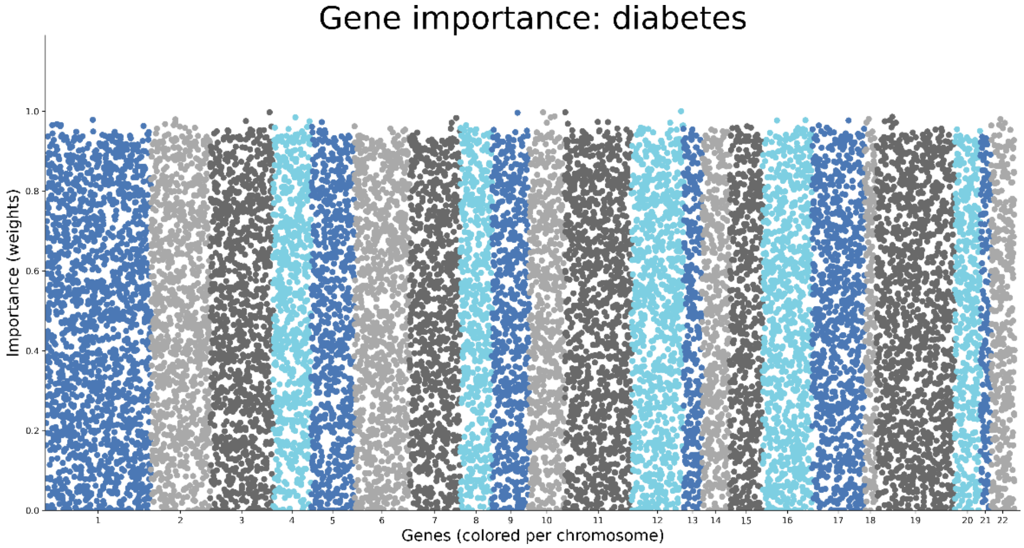

Supplementary Figure 29. Manhattan plot for genes important for the network for separating between cases and controls for diabetes. Although we are underpowered, there seems to be more distinction between genes than for the CAD and AF.

Supplementary Note 5. Rotterdam Study

5.1 Eye color – blue vs rest

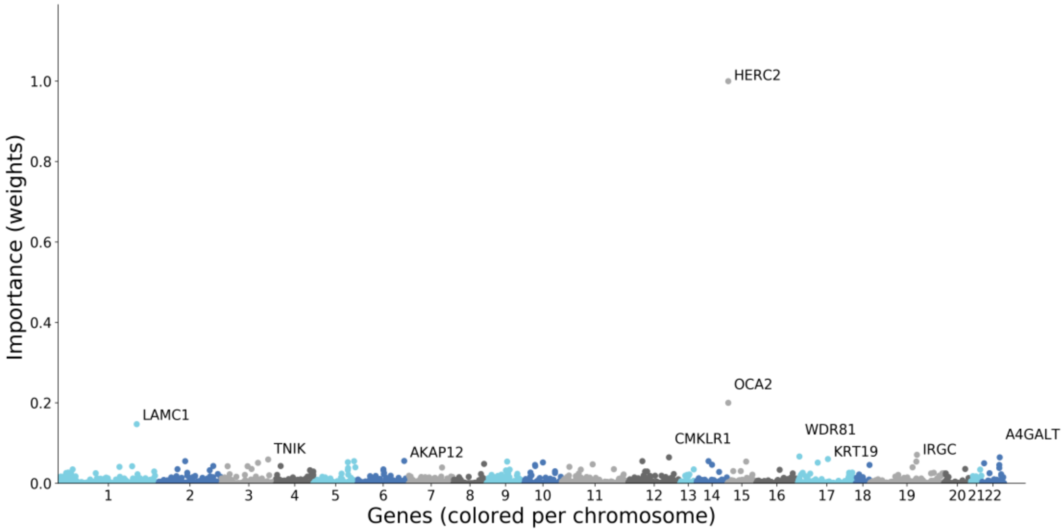

Supplementary Figure 30. Manhattan plot for genes important for the network for separating between blue and other color eyes.

Supplementary Method 6. Estimating upper bound classification accuracy

Imagine the following hypothetical situation: for every person in our dataset there exists a monozygotic twin. In our experiment we use the monozygotic twin for the prediction of a phenotype.
Our first phenotype of interest is schizophrenia. It has a concordance rate = 0.5 in monozygotic twins and a prevalence of roughly 1%. We can use these to construct a confusion matrix for our phenotype of interest:

What if the first twin is schizophrenic?

- The chance that the twin in our dataset is also diseased is simply the concordance rate, 41%.
- The chance that we misclassify the twin in the real world as a false positive is 1-concordance rate * 100% = 59 %

What if the first twin is healthy?

- The chance that the second twin is healthy is higher than the 1-prevalence. Thus, bigger than 99% since the twins share genetically the same code. Let's make this 100% since we are interested in the maximum performance.
- The chance that we misclassify the twin as a false positive is smaller than the prevalence so a maximum of 2%.

Resulting in the following confusion matrix:

True positive (concordance rate)<br>0.41 * 4969 = **2037**	False Positive (1- concordance rate)<br>(1-0.41) * 4969 = 2931
False negative (< prevalence)<br>0.02* 6245 = 125	True negative (> (1-prevalence))<br>(1-0.02) * 6245 = **6120**

Maximum Accuracy $= \frac{\text{predicted correctly}}{\text{Total}}$

$$= \frac{(\text{concordance}*\text{cases})+\text{controls}(1-\text{prevalence})}{\text{Total cases and controls}}$$

$$= \frac{0.41*4939+(1-0.02)6245}{4969 + 6245} = 0.73$$

The maximum accuracy I can reach with this distribution of cases and controls is ~73%.

This is the perfect classifier if we purely use genetic data. No machine learning or deep learning model can do better without adding (environmental) information.

The confusion matrix can be used to calculate more metrics such as sensitivity, specificity and F_1-score. The sensitivity and specificity can be plotted in the ROC curve to control for overfitting.

6.1 Overview of the upper bound classification accuracies in this study

Table with the upper bound accuracy according to the thought experiment in Supplementary 6.1. For some phenotypes such as hair color, the estimate might be less reliable due to migration (i.e., black hair color was natively not this prevalent in the UK). This methodological approach does not take in account migration, because of this the maximum accuracy might be underestimated for hair color.

Trait	Phenotype	Concordance	Prevalence	Cases	Controls	Max. Accuracy	Ref Conc.	Ref Prev.
**Eye color**	Blue	0.98	0.61	4041	2250	0.85	9	9
**Hair color**	Red	0.94	0.08	1734	1727	0.93	10	9
	Black	0.94	0.04*	3762	3753	0.95	11	11
	Brown	0.94	0.78	31892	31947	0.86	11	11
	Blond	0.94	0.41	4501	4518	0.76	11	11
**Bipolar**	Case	0.43	0.02	343	347	0.71	12	13
**Atrial fibrillation**	Case	0.22	0.03	192	194	0.60	14	15
**CAD**	Case	0.40	0.03	1563	1600	0.69	16	15
**Dementia**	Case	0.67	0.02	139	142	0.83	17	13
**Asthma**	Case	0.50	0.12	4229	4214	0.69	18	19
**Diabetes type II**	Case	0.87	0.05	2557	2555	0.91	20	21
**Breast cancer**	Case	0.28	0.01	1070	1082	0.64	22	23
**Schizophrenia**	Case	0.41	0.02	4969	6245	0.73	24	25, 13
**Schizophrenia**	Case	0.65	0.02	4969	6245	0.83	24	25, 13

*Supplementary Table 6 Overview of the estimated upper bound of the accuracy for the datasets used in this study. This table contains all relevant statistics used for this estimate (See supplementary 6). The monozygotic twin concordance and prevalence were obtained from literature. *This methodological approach does not take in account migration, because of this the upper bound accuracy might be underestimated.*

Supplementary Discussion 7. Deletion of predictive connections

In this experiment we evaluate the performance while deleting the connections to the most predictive features. We evaluated this for two widely different phenotypes, eye color, where HERC2 and OCA2 are the main contributors to the prediction of blue eye color and schizophrenia, a polygenic disease with numerous genes contributed to the prediction. As expected, the curves in Supplementary Figures 31 & 32 are different for the two phenotypes. The prediction for eye color deteriorates quickly, even by only deleting the connections to the HERC2 gene, while the predictive performance of schizophrenia is relatively unaffected even if the connections to the top thousand predictive genes are deleted.

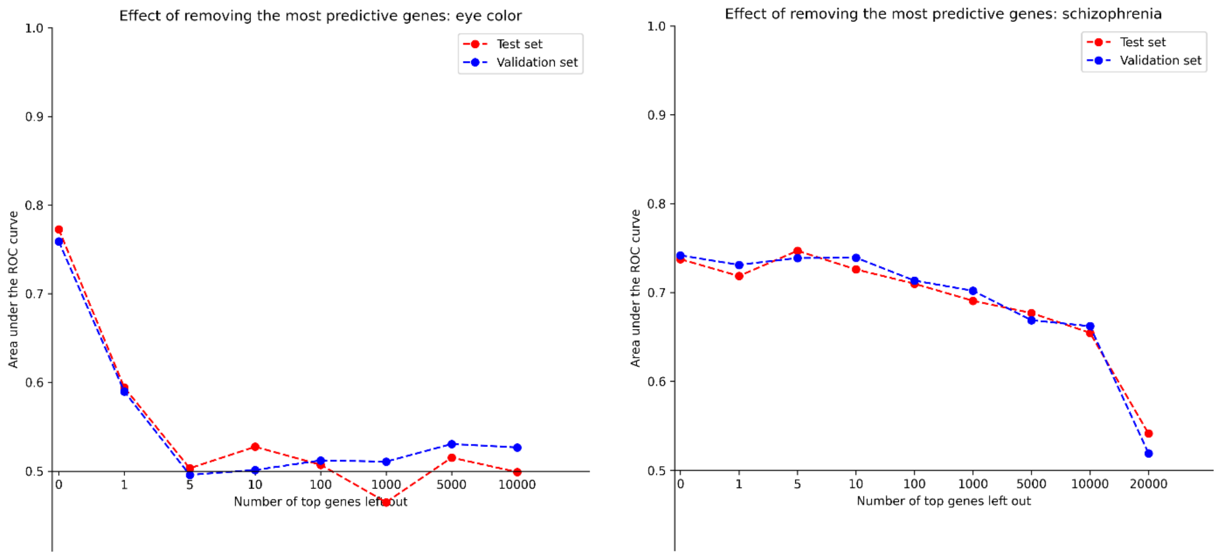

Supplementary Figure 31 & 32. Performance of the network while removing up to 20 000 connections. The genes are sorted by contribution/importance and deleted in this order, with connections to most important genes first deleted.

Supplementary Discussion 8. Does prior knowledge improve performance?

Embedding prior knowledge allows us to interpret the weights in the neural networks. One could speculate that embedding prior knowledge could also help in guiding training, resulting in better converged networks with better performance than networks without prior knowledge.

This experiment is designed to test the hypothesis: '*Embedding prior knowledge (gene annotations) in the neural network results in a network with a better performance than an equivalent network without prior knowledge'*.

In this experiment, we used GenNet networks identical to the network used in the experiments of Supplementary Table 2.1 with gene annotations as prior knowledge embedded in the network. The randomly connected networks are obtained by randomly shuffling the connectivity matrix in the horizontal direction. In the resulting network, all SNPs are randomly connected to nodes in the next layer (formerly known as the gene layer, now uninterpretable). All SNPs are thus connected to a random node and these nodes are connected to the output. Aside from this, the networks are equal in all aspects, they have the same number of trainable parameters (see supplementary 8.1 Gene network) and all networks are trained with GenNet's default hyperparameters (Adam with learning rate of 0.01 and L1 penalty of 0.01). We trained ten differently randomly connected networks and compared those to an equal number of GenNet gene networks for the Rotterdam Study and Sweden Schizophrenia. Due to limitations in resources, number of phenotypes and time constraints we decided to train six network per phenotype in the UK biobank (three shuffled and three regular). In total 112 networks were trained for this experiment.

Inspecting Supplementary Table 7 shows that embedding prior knowledge in the neural network architecture does not lead to significantly better performance for all phenotypes. The results are inconclusive. For example, for red hair color we observe a non-significant improvement but this is not maintained for predicting black or blond hair color. The randomly connected network performs significantly better than a network with prior knowledge for schizophrenia but we find the opposite for predicting blue eye color in the Rotterdam color, GenNet significantly outperforms a randomly connected network. Thus, in general we cannot conclude that prior knowledge neither improves or deteriorates the performance.

**Dataset (type)**	**Trait**	**Subjects & phenotype**		**Number of runs**	**AUC randomly connected network (val)**	**AUC randomly connected network (test)**	**AUC GenNet (val)**	**AUC GenNet (test)**	**P-value Difference AUC (test)**
		**Class I**	**Class II**						
Rotterdam (genotype array)	Eye color	4041 Blue	2250 Other	10	0.754±0.007	0.762±0.007	0.761±0.004	**0.771 ± 0.002**	6.54 × 10-3
UK Biobank (exome)	Hair color	4501 Blond	4518 Other	3	0.658±0.004	0.652±0.008	0.623±0.024	0.623±0.020	0.250
		15684 Dark brown	15918 Other	3	0.615±0.011	0.624±0.008	0.608±0.010	0.612±0.006	0.359

		1734<br>Red	1727<br>Other	3	0.837±0.021	0.834±0.017	0.907±0.018	0.900±0.022	0.084
		16208<br>Light<br>brown	16029<br>Other	3	0.601±0.002	0.605±0.003	0.582±0.005	0.593±0.004	0.089
		3762<br>Black	3753<br>Other	3	0.831±0.003	0.818±0.004	0.820±0.010	0.800±0.011	0.068
	Atrial<br>fibrillation	192<br>Cases	194<br>Controls	3	0.513±0.047	0.483±0.063	0.466±0.048	0.554±0.032	0.331
	Coronary<br>Artery<br>Disease	1563<br>Cases	1600<br>Controls	3	0.537±0.009	0.547±0.008	0.522±0.029	0.526±0.007	0.151
	Diabetes	2557<br>Cases	2555<br>Controls	3	0.544±0.003	0.545±0.002	0.527±0.046	0.524±0.020	0.201
	Dementia	139<br>Cases	142<br>Controls	3	0.466±0.033	0.492±0.069	0.531±0.069	0.464±0.083	0.677
	Allergies	10242<br>Cases	10187<br>Controls	3	0.522±0.01	0.513±0.004	0.489±0.007	0.505±0.005	0.283
	Breast<br>cancer	1070<br>Cases	1082<br>Controls	3	0.529±0.017	0.525±0.015	0.529±0.012	0.539±0.020	0.618
	Asthma	4229<br>Cases	4214<br>Controls	3	0.534±0.009	0.531±0.005	0.507±0.010	0.51±0.020	0.230
Sweden<br>(exome)	Schizophre<br>nia	4969<br>Cases	6245<br>Controls	10	0.755±0.01	**0.755±0.006**	0.740±0.004	0.737±0.006	2.50 × 10-4

Supplementary Table 7. Overview of the experiments to determine if prior knowledge, aside from making the network interpretable, also improves performance. Per phenotype, the mean and standard deviations of the AUC over three runs for the UK biobank and ten runs for the other two datasets are shown. Significant better performance in a two-sided student's t-test is emphasized.

Supplementary Note 9. GenNet architectures

9.1 All architectures: summary table

Dataset (type)	Number of input variants	Network type	Layer<br>N of nodes<br>(n connections)					Total number of trainable parameters
Rotterdam (genotype array)	113,241 input variants	Gene network	Gene layer<br>16628<br>(129869)	Out<br>1<br>(16629)				146,498
		Pathway network	Gene layer<br>16628<br>(129869)	Pathway 1<br>337<br>(21325)	Pathway2<br>44<br>(374)	Pathway 3<br>6<br>(50)	Out<br>1<br>(7)	151,625
		GTEx expression networks	Gene layer<br>16356<br>(126716)	Tissue layer<br>53<br>(96327)	Out<br>1<br>(54)			223,097
		ImmGen expression networks	Gene layer<br>16628<br>(126716)	Cell layer<br>292<br>(428174)	Out<br>1<br>(293)			555,183
UK Biobank (exome)	6,986,636 input variants	Gene network	Gene layer<br>15827<br>(6661236)	Out<br>1<br>(15828)				6,677,064
		Pathway network<br>GTEx expression networks	Gene layer<br>15827<br>(6661236)	Pathway 1<br>337<br>(23550)	Pathway2<br>44<br>(374)	Pathway 6<br>(50)	Out<br>1<br>(7)	6,685,217
		GTEx expression networks	Gene layer<br>21476<br>(6668279)	Tissue layer<br>53<br>(80249)	Out<br>1<br>(54)			6,748,582
		ImmGen expression networks	Gene layer<br>21476<br>(6668279)	Cell layer<br>292<br>(316342)	Out<br>1<br>(293)			6,984,914
Sweden (exome	1,288,701 input variants	Gene network	Gene layer<br>21390<br>(1310091)	Out<br>1<br>(21391)				1,331,482
		Pathway network	Gene layer<br>21390<br>(1310091)	Pathway 1<br>330<br>(30851)	Pathway2<br>44<br>(432)	Pathway 3<br>6<br>(50)	Out<br>1<br>(7)	1,341,431
		GTEx brain expression networks	Gene layer<br>21390<br>(1310091)	Gene layer<br>23765<br>(1312466)	Tissue layer<br>13<br>(27253)	Out<br>1<br>(14)		1,339,733
		GTEx expression networks	Gene layer<br>21390<br>(1310091)	Tissue layer<br>53<br>(109458)	Out<br>1<br>(54)			1,421,978

ImmGen expression networks	Gene layer 23765 (1312466)	Tissue layer 292 (468421)	Out 1 (293)	1,781,180

Supplementary Table 8. Overview of the architectures used in this study. With the number of nodes in each layer and the number of weights/connections between brackets. The last column contains the number of trainable parameters for the architecture. The networks are phenotype independent, but do differ per dataset since each dataset contains different input variants.

9.2 Prior knowledge

Gene Layer: all SNPs are annotated using Annovar (see 9.3 and bibliography). [3] Using regular expression, all genes are filtered and SNPs without gene annotations are dropped. The complete pipeline can be found in:

https://github.com/ArnovanHilten/GenNet/blob/master/jupyter_notebooks/2_Define_connection_masks.ipynb

Pathway layer: All genes used in the gene layer are annotated using GeneSCF[26] and connected to their subsequent pathways using the KEGG website (https://www.genome.jp/kegg/pathway.html).

ImmGen and GTEx: First we obtained the t-statistic matrices from Finucane et al. (2018) [5]. In their work Finucane et al computed for each gene a t-statistic for specific expression in the focal tissue. The 10% of genes with the highest t-statistic were assigned to the gene set corresponding to the focal tissue. The 10% threshold was chosen because it gave the most significant p-values in two of their datasets. In their evaluation of this parameter, they showed that their choice was valid, results did not change when using a 5% or 20% threshold (their supplementary figures 2a-c). Following their approach, we connected for each tissue the genes in the top 10% t-statistic tot that tissue.

9.3 URLS

Trained GenNet architectures deposit:

https://github.com/ArnovanHilten/GenNet_ModelZoo

Software

Annovar

https://annovar.openbioinformatics.org/en/latest/ (free to use, sign-up required)

GeneSCF:

https://github.com/genescf

Data

ImmGen layer:

https://alkesgroup.broadinstitute.org/LDSCORE/LDSC_SEG_ldscores/tstats/ImmGen.tstat.tsv

GTEx expression layer:

https://alkesgroup.broadinstitute.org/LDSCORE/LDSC_SEG_ldscores/tstats/GTEx.tstat.tsv

Brain cell expression layer:

https://alkesgroup.broadinstitute.org/LDSCORE/LDSC_SEG_ldscores/tstats/GTEx_brain.tstat.tsv

scRNA-seq data (FUMA) layer:

https://github.com/Kyoko-wtnb/FUMA_scRNA_data

Supplementary Method 10. Regression

For regression tasks, mean squared error is used as a loss function in combination with ReLu activations. Using the UK biobank WES data, the explained variance for height was 31% using linear regression, whereas the network achieved 9% explained variance. We only tested the technical implementation, without any optimization. We expect the network to outperform or at least match linear regression with optimization, since the network has more modelling capabilities than linear regression. In the framework the type of task (regression or classification) is automatically determined using the phenotype labels.

Supplementary References

1. van Hilten, A. *et al.* ArnovanHilten/GenNet: Release GenNet 1.4. (2021) doi:10.5281/ZENODO.5151527.
2. Wray, N. R., Yang, J., Goddard, M. E. & Visscher, P. M. The genetic interpretation of area under the ROC curve in genomic profiling. *PLoS Genet.* **6**, (2010).
3. Wang, K., Li, M. & Hakonarson, H. ANNOVAR: functional annotation of genetic variants from high-throughput sequencing data. *Nucleic Acids Res.* **38**, e164–e164 (2010).
4. Kanehisa, M. & Goto, S. KEGG: kyoto encyclopedia of genes and genomes. *Nucleic Acids Res.* **28**, 27–30 (2000).
5. Finucane, H. K. *et al.* Heritability enrichment of specifically expressed genes identifies disease-relevant tissues and cell types. *Nat. Genet.* **50**, 621–629 (2018).
6. Heng, T. S. P., Painter, M. W., Immunological, T. & Project, G. The Immunological Genome Project : networks of gene expression in immune cells. **9**, 1091–1094 (2008).
7. Nousbeck, J. *et al.* A mutation in a skin-specific isoform of SMARCAD1 causes autosomal-dominant adermatoglyphia. *Am. J. Hum. Genet.* **89**, 302–307 (2011).
8. Han, J. *et al.* A genome-wide association study identifies novel alleles associated with hair color and skin pigmentation. *PLoS Genet.* **4**, (2008).
9. Katsara, M. A. & Nothnagel, M. True colors: A literature review on the spatial distribution of eye and hair pigmentation. *Forensic Sci. Int. Genet.* **39**, 109–118 (2019).
10. Matheny, A. P. & Dolan, A. B. Changes in Eye colour during early childhood: Sex and genetic differences. *Ann. Hum. Biol.* **2**, 191–196 (1975).
11. Morgan, M. D. *et al.* The genetic architecture of hair colour in the UK population. *bioRxiv* (2018) doi:10.1101/320267.
12. Kieseppä, T., Partonen, T., Haukka, J., Kaprio, J. & Lönnqvist, J. High concordance of bipolar I disorder in a nationwide sample of twins. *Am. J. Psychiatry* **161**, 1814–1821 (2004).
13. Prince, M. *et al.* No health without mental health. *Lancet* **370**, 859–877 (2007).
14. Christophersen, I. E. *et al.* Familial aggregation of atrial fibrillation: A study in danish twins. *Circ. Arrhythmia Electrophysiol.* **2**, 378–383 (2009).
15. Bhatnagar, P., Wickramasinghe, K., Wilkins, E. & Townsend, N. Trends in the epidemiology of cardiovascular disease in the UK. *Heart* **102**, 1945–1952 (2016).
16. Zdravkovic, S. *et al.* Heritability of death from coronary heart disease: A 36-year follow-up of 20 966 Swedish twins. *J. Intern. Med.* **252**, 247–254 (2002).
17. Gatz, M. *et al.* Heritability for Alzheimer's disease: The study of dementia in Swedish twins. *Journals Gerontol. - Ser. A Biol. Sci. Med. Sci.* **52**, 117–125 (1997).

18. Strachan, D. P., Wong, H. J. & Spector, T. D. Concordance and interrelationship of atopic diseases and markers of allergic sensitization among adult female twins. *J. Allergy Clin. Immunol.* **108**, 901–907 (2001).

19. Asthma statistics | British Lung Foundation. https://statistics.blf.org.uk/asthma.

20. Willemsen, G. *et al.* The Concordance and Heritability of Type 2 Diabetes in 34,166 Twin Pairs From International Twin Registers: The Discordant Twin (DISCOTWIN) Consortium. *Twin Res. Hum. Genet.* **18**, 762–771 (2015).

21. Bycroft, C. *et al.* The UK Biobank resource with deep phenotyping and genomic data. *Nature* **562**, 203–209 (2018).

22. Möller, S. *et al.* The heritability of breast cancer among women in the nordic twin study of cancer. *Cancer Epidemiol. Biomarkers Prev.* **25**, 145–150 (2016).

23. Forman, D. *et al.* Cancer prevalence in the UK: Results from the EUROPREVAL study. *Ann. Oncol.* **14**, 648–654 (2003).

24. Cardno, A. G. & Gottesman, I. I. Twin studies of schizophrenia: From bow-and-arrow concordances to star wars Mx and functional genomics. *Am. J. Med. Genet. - Semin. Med. Genet.* **97**, 12–17 (2000).

25. McGrath, J., Saha, S., Chant, D. & Welham, J. Schizophrenia: A concise overview of incidence, prevalence, and mortality. *Epidemiol. Rev.* **30**, 67–76 (2008).

26. Subhash, S. & Kanduri, C. GeneSCF: a real-time based functional enrichment tool with support for multiple organisms. *BMC Bioinformatics* **17**, 365 (2016).
