# Supplementary material for: GenNet framework: interpretable deep learning for predicting phenotypes from genetic data
Source: Commun Biol. 2021 Sep 17;4:1094. doi: 10.1038/s42003-021-02622-z (PMC8448759; doi:10.1038/s42003-021-02622-z)
Supplement: Supplementary file 3 — Reporting Summary [file 42003_2021_2622_MOESM3_ESM.pdf]

nature portfolio

Corresponding author(s): Arno van Hilten

Last updated by author(s): Jul 27, 2021

Reporting Summary

Nature Portfolio wishes to improve the reproducibility of the work that we publish. This form provides structure for consistency and transparency in reporting. For further information on Nature Portfolio policies, see our Editorial Policies and the Editorial Policy Checklist.

Statistics

For all statistical analyses, confirm that the following items are present in the figure legend, table legend, main text, or Methods section.

n/a	Confirmed	
☐	☒	The exact sample size (*n*) for each experimental group/condition, given as a discrete number and unit of measurement
☐	☒	A statement on whether measurements were taken from distinct samples or whether the same sample was measured repeatedly
☐	☒	The statistical test(s) used AND whether they are one- or two-sided<br>*Only common tests should be described solely by name; describe more complex techniques in the Methods section.*
☒	☐	A description of all covariates tested
☐	☒	A description of any assumptions or corrections, such as tests of normality and adjustment for multiple comparisons
☐	☒	A full description of the statistical parameters including central tendency (e.g. means) or other basic estimates (e.g. regression coefficient) AND variation (e.g. standard deviation) or associated estimates of uncertainty (e.g. confidence intervals)
☒	☐	For null hypothesis testing, the test statistic (e.g. *F*, *t*, *r*) with confidence intervals, effect sizes, degrees of freedom and *P* value noted<br>*Give P values as exact values whenever suitable.*
☒	☐	For Bayesian analysis, information on the choice of priors and Markov chain Monte Carlo settings
☐	☒	For hierarchical and complex designs, identification of the appropriate level for tests and full reporting of outcomes
☐	☒	Estimates of effect sizes (e.g. Cohen's *d*, Pearson's *r*), indicating how they were calculated

Our web collection on statistics for biologists contains articles on many of the points above.

Software and code

Policy information about availability of computer code

Data collection	No data was collected in this study
Data analysis	The GenNet framework is open-source project under the Apache-2.0 License. An exhaustive list of all the dependencies can be found in the requirements file on Github. The most important dependencies for the framework are:<br>Tensorflow (1.12.0 and higher), numpy (1.17.1), pandas(0.25.1), h5py (2.10.0), tables(3.5.1), scikit-learn (0.22.1), scipy(1.4.1).<br><br>Prior knowledge used to construct network architecture was obtained using ANNOVAR (at https://annovar.openbioinformatics.org/) GeneSCF (https://github.com/genescf), GTEx (https://gtexportal.org/home/datasets), and t-score statics derived by Finucane et al (2018) (https://alkesgroup.broadinstitute.org/LDSCORE/). All freely available.

For manuscripts utilizing custom algorithms or software that are central to the research but not yet described in published literature, software must be made available to editors and reviewers. We strongly encourage code deposition in a community repository (e.g. GitHub). See the Nature Portfolio guidelines for submitting code & software for further information.

Data

Policy information about availability of data

All manuscripts must include a data availability statement. This statement should provide the following information, where applicable:

- Accession codes, unique identifiers, or web links for publicly available datasets
- A description of any restrictions on data availability
- For clinical datasets or third party data, please ensure that the statement adheres to our policy

Code to run and generate data for the simulations are publicly available on GitHub. The genetic and phenotypic UK Biobank data are available upon application to

the UK Biobank (https://www.ukbiobank.ac.uk/). Access to the Sweden-Schizophrenia Exome Sequencing study can be requested on DBGaP (https://www.ncbi.nlm.nih.gov/gap/) (dbGaP phs000473.v2.p2). Data from the Rotterdam Study are not publicly available due to informed consent and legal restrictions (e.g. GDPR law in the EU). However, specific requests for access to the data can be addressed to the Rotterdam Study Management Team that assesses the proposals and adjudicates access - in line with national and international regulations - on a case-by-case basis.

Field-specific reporting

Please select the one below that is the best fit for your research. If you are not sure, read the appropriate sections before making your selection.

☒ Life sciences ☐ Behavioural & social sciences ☐ Ecological, evolutionary & environmental sciences

For a reference copy of the document with all sections, see nature.com/documents/nr-reporting-summary-flat.pdf

Life sciences study design

All studies must disclose on these points even when the disclosure is negative.

Sample size	We used all available subject data from the UK Biobank, Rotterdam study and Sweden-Schizophrenia Exome Sequencing study with the relevant phenotypes. For every phenotype an equal number of cases and controls were randomly sampled.
Data exclusions	Related subjects were excluded from the validation and test set.
Replication	The dataset is split in a train, validation and a test set (ratio of 60/20/20). Related cases, and cases with related controls, (kinship > 0.0625) are all in the training set. The model is trained on the training set, validated on the validation set and only tested once on the held-out test set.
Randomization	Subjects were randomly assigned to the different sets.
Blinding	This was not a randomized control trial, thus it did not need blinding

Reporting for specific materials, systems and methods

We require information from authors about some types of materials, experimental systems and methods used in many studies. Here, indicate whether each material, system or method listed is relevant to your study. If you are not sure if a list item applies to your research, read the appropriate section before selecting a response.

Materials & experimental systems

n/a	Involved in the study	
☒	☐	Antibodies
☒	☐	Eukaryotic cell lines
☒	☐	Palaeontology and archaeology
☒	☐	Animals and other organisms
☒	☐	Human research participants
☒	☐	Clinical data
☒	☐	Dual use research of concern

Methods

n/a	Involved in the study	
☒	☐	ChIP-seq
☒	☐	Flow cytometry
☒	☐	MRI-based neuroimaging
